# Topological phononics arising from fluid-solid interactions

**DOI:** 10.1038/s41467-022-33896-4

**Published:** 2022-10-17

**Authors:** Xiaoxiao Wu, Haiyan Fan, Tuo Liu, Zhongming Gu, Ruo-Yang Zhang, Jie Zhu, Xiang Zhang

**Affiliations:** 1 Faculties of Sciences and Engineering, The University of Hong Kong, Hong Kong, China; 2 Quantum Science and Technology Center and Advanced Materials Thrust, The Hong Kong University of Science and Technology (Guangzhou), Nansha, Guangzhou, 511400 Guangdong China; 3 Department of Mechanical Engineering, The Hong Kong Polytechnic University, Hung Hom, Kowloon, Hong Kong, China; 4 Key Laboratory of Noise and Vibration Research, Institute of Acoustics, Chinese Academy of Sciences, Beijing, 100190 China; 5 Institute of Acoustics, School of Physics Science and Engineering, Tongji University, Shanghai, 200092 China; 6 Department of Physics, The Hong Kong University of Science and Technology, Clear Water Bay, Kowloon, Hong Kong, China

**Keywords:** Topological matter, Acoustics, Structure of solids and liquids

## Abstract

Nontrivial band topologies have been discovered in classical systems and hold great potential for device applications. Unlike photons, sound has fundamentally different dynamics and symmetries in fluids and solids, represented as scalar and vector fields, respectively. So far, searches for topological phononic materials have only concerned sound in either fluids or solids alone, overlooking their intricate interactions in “mixtures”. Here, we report an approach for topological phononics employing such unique interplay, and demonstrate the realization of type-II nodal rings, elusive in phononics, in a simple three-dimensional phononic crystal. Type-II nodal rings, as line degeneracies in momentum space with exotic properties from strong tilting, are directly observed through ultrasonic near-field scanning. Strongly tilted drumhead surface states, the hallmark phenomena, are also experimentally demonstrated. This phononic approach opens a door to explore topological physics in classical systems, which is easy to implement that can be used for designing high-performance acoustic devices.

## Abstract (web summary)

Fluid-solid interaction, long investigated, is mostly neglected in topological acoustics. Here the authors find that it can give rise to intriguing topological phenomena in simple phononic crystals due to intrinsic differences between sound in fluid and solid.

## Introduction

Nontrivial band topologies, stemming from nodal points of quantum condensed matters where energy bands intersect each other in the reciprocal space^1,2^, have been discovered in photonic and phononic systems^3,4^. Manifested by exotic transport of photons^5,6^ and sound waves^7–9^, they hold great potential for applications^10–20^ such as lasing^15,16^, sensing^17,18^, and energy harvesting^19,20^. Due to their close relationship, searches for topological phononic systems are often inspired by the counterparts in photonics^4^ and only concern sound in either fluids or solids alone. As a mechanical wave^21^, sound propagates as a perturbation of density in fluids and elastic displacements in solids, and it is hence represented by a scalar field and vector field, respectively. This fundamental point leads to intrinsic differences in the dynamics and symmetries for sound in fluids and solids, a characteristic absent in photonics^22^. However, such intrinsic differences and their possible interactions have yet to be considered in the development of topological phononics, even for the underwater environment where the interactions can become considerable^23,24^.

In fact, among the band topologies besides touching at a single point, which forms a Dirac node^25^ or Weyl node^26^, the nodal points can also form line degeneracies along a closed loop, so-called nodal lines^27,28^, with geometric configurations such as nodal rings^29^, nodal chains^30^ and nodal links^31^. Originally perceived to be mimicking high-energy particles dictated by Lorentz invariance, however, an extra tilting can exist for the nodal points in the bands of periodic systems^32^. When the tilting becomes strong enough such that it cannot be adiabatically removed^33^, unconventional type-II Dirac points^14,34^, Weyl points^35,36^, and nodal rings^37,38^ arise, distinguished by finite density of states at the node energy and strong anisotropic transport properties compared with their type-I counterparts^39–41^. To date, there is still no realization for type-II nodal rings in phononics, which seems to require more tortuous designs than already very complicated realizations of type-II Dirac or Weyl points using phononic crystals^11,14^.

In this article, we propose and experimentally demonstrate type-II nodal rings for sound with a unique approach developed for topological phononics. The approach utilizes the different wave dynamics and symmetry representations of sound in fluids and solids, specifically, the interplay of the scalar nature of sound in fluids and its vectorial nature in solids, a feature absent for photons. With this approach, type-II nodal rings are realized in a simple three-dimensional (3D) phononic crystal, which is identical perforated metallic plates immersed in water. Arising from the interaction between waterborne sound and elastic modes of the solid plates, the type-II nodal rings are revealed through ultrasonic near-field scanning. Strongly tilted drumhead surface states (DSSs) stemming from the type-II nodal rings are also experimentally observed. Nodal chains also emerge in the simple structure. Our study demonstrates that the interaction in phononic crystals made of fluid-solid “mixtures” (unit cells comprising both fluid and solid components), previously often disregarded for simplicity when searching topological materials in phononics, can lead to rich phenomena unattainable for solely fluid-borne or solid-borne sound. Thereby it provides a novel platform for exploring unique topological physics and acoustic applications.

## Results

### Type-II nodal rings arising from fluid-solid interactions

**Fig. 1:**
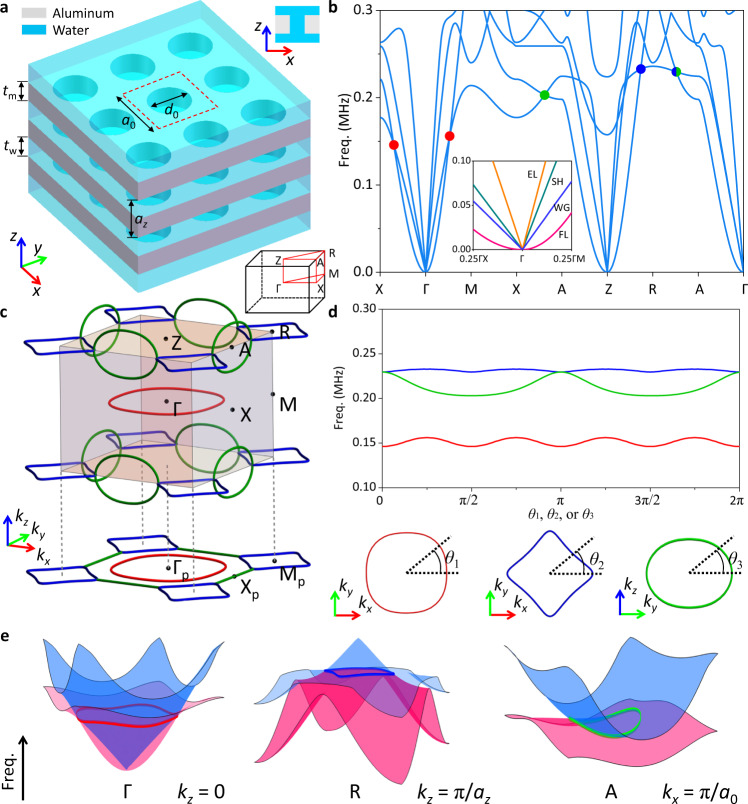
Type-II nodal rings induced by fluid-solid interaction. **a** Perspective and cross-sectional views of the 3D phononic crystal, formed with identical aluminum plates with circular through holes immersed in water. The phononic crystal belongs to the space group P4/mmm (No. 123). Inset: schematic of the first Brillouin zone (FBZ). The red dashed square indicates a single unit cell (*z* direction not shown). The in-plane lattice constant *a*_0_ = 3 mm, the diameter of holes *d*_0_ = 2 mm, the thickness of plates *t*_m_ = 2 mm, the separation of plates *t*_w_ = 2 mm, corresponding to out-of-plane lattice constant *a*_z_ = *t*_m_ + *t*_w_ = 4 mm. **b** Calculated band structure along high-symmetry directions in FBZ. The dots indicate the crossings of the 1st and 2nd bands, with different colors indicating different nodal rings. Inset: Enlarged view of the bands around Γ point, where the four bands are identified as flexural Lamb (FL) modes, waterborne guided (WG) modes, shear horizontal (SH) modes, and extensional Lamb (EL) modes, respectively. **c** Distribution of the nodal rings in the FBZ. The colors of the nodal rings the same as the corresponding dots in (**b**). **d** Spectral variations of the three nodal rings. **e** 3D band structures of the first two modes on specified cross sections of the FBZ. Nodal rings formed by their crossings are denoted.

We start from considering a 3D phononic crystal immersed in a fluid (Fig. 1a). The phononic crystal comprises identical metallic plates separated by the fluid, each plate perforated with a square lattice of circular through holes. Here, we adopt aluminum and water for demonstration purpose. Water, unlike air, have comparable acoustic impedance with aluminum, substantially facilitating fluid-solid interaction. The first four bands of the phononic crystal originate from the three lowest plate modes and the waterborne sound. The calculated band structure (Fig. 1b) along the high-symmetry directions of the first Brillouin zone (FBZ) features their emergence from zero frequency at Γ point. The 1st band intersects with the 2nd band on high-symmetry planes of the FBZ. Their touching points, as indicated by the red, blue, and green dots, form different nodal rings, respectively. Moreover, these touching points are all gapped slightly away from corresponding high-symmetry planes in the reciprocal space, which are the characteristics of nodal rings (see Supplementary Note 1). The calculated distribution of the nodal rings (Fig. 1c) in the FBZ further reveal that the blue and green nodal rings are chained together, forming nodal chains. Detailed shapes and frequency variances of the nodal rings are given (Fig. 1d). According to the band slopes around the nodal rings, the red and blue nodal rings are type-II nodal rings, while the green nodal rings are hybrid nodal rings (Fig. 1e). The hybrid green nodal rings have a relatively large frequency dispersion (Δ*ω*/*ω*_mid_ ~ 12.3%, with Δ*ω* being the frequency variation and *ω*_mid_ being the middle of frequencies), while the type-II red and blue nodal rings have smaller frequency dispersions (Δ*ω*/*ω*_mid_ ~ 6.6% and ~ 1.4% for the red and blue ones). Additional nodal rings may also appear between the 2nd and higher bands, but they are all inside the sound cone (bulk acoustic waves in water) when projected along the *z* direction, out of our interest.

### Theoretical modeling of intrinsic dynamics

**Fig. 2:**
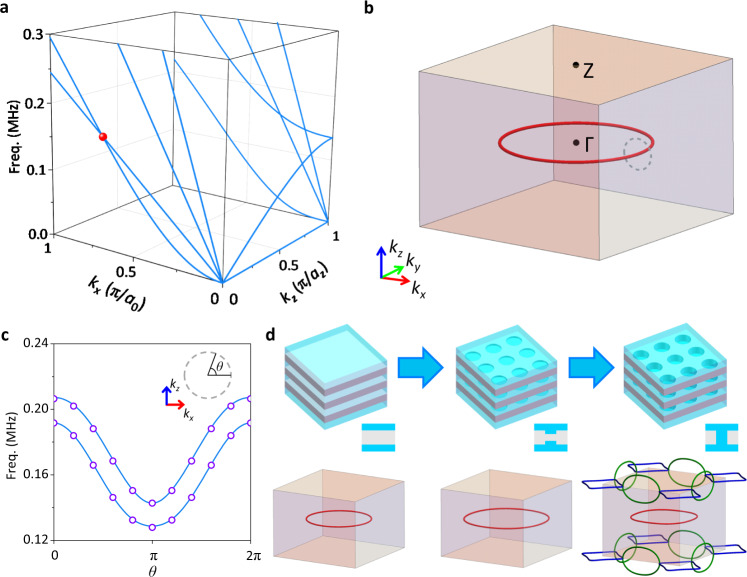
Origins and analysis of the nodal rings. **a** Band structure of periodic metallic plates without perforations. **b** Distribution of the ideal type-II nodal ring for (**a**). No other nodal rings exist. **c** Band structure on the dashed loop denoted in (**b**). The radius of the circular loop is 0.1π/*a*_0_. Solid lines and hollow dots respectively denote the results from full-wave simulations and the effective Hamiltonian. **d** Evolution of the nodal rings, from uniform plates to plates with blind holes and finally through holes.

Notably, the nodal rings originate from the fluid-solid interaction, since transverse components of the plates’ displacements cannot be ignored (see Supplementary Note 2). To clarify this point, we first consider band dispersions near Γ point (inset in Fig. 1(b)), around which the first four bands can be classified as the lowest-order waterborne guided (WG) mode and the three lowest-order plate modes; the flexure Lamb (FL) mode, the extensional Lamb (EL) mode, and the shear horizontal (SH) mode (see Supplementary Note 3 for their mode profiles near Γ point). The WG mode and FL mode have opposite parities with respect to the mirror symmetry *M*_*z*_ (*z* → −*z*) and are decoupled when *k*_*z*_ = 0 (see Supplementary Note 15). Far away from the Γ point, the hybridizations between these modes can be significant, so that the bands cannot be easily identified. To illuminate the emergence of the nodal rings, we consider the case that periodic metallic plates are arranged in the same manner but without any perforations. The calculated bands still feature the touching point between the 1st and 2nd bands on *k*_z_ = 0 plane (Fig. 2a). In this case, around Γ point with *k*_*z*_ = 0, we derive the dispersions based on the transfer matrix method (see Supplementary Note 9)

1 $$\begin{array}{c}{\omega }_{{{{{{\rm{WG}}}}}}}={c}_{w}{k}_{r}\\ {\omega }_{{{{{{\rm{FL}}}}}}}=\sqrt{\frac{D}{{\rho }_{m}{t}_{m}+{\rho }_{w}{t}_{w}}}{k}_{r}^{2},\end{array}$$

where ${k}_{r}=\sqrt{{k}_{x}^{2}+{k}_{y}^{2}}$, *D* and *ρ*_*m*_ are the bending stiffness and density of the plates, *c*_w_ and *ρ*_*w*_ are the sound speed and density of the background fluid (water). Therefore, in the long-wavelength limit, for the FL mode, a quadratic asymptotic behavior *ω*_FL_ ~ *k*_*r*_^2^ is observed, which is similar to the case of flexural waves on a free-standing thin plate. In contrast, for the WG mode coinciding with sound cone, a typical linear asymptotic behavior *ω*_WG_ ~ *k*_*r*_ is observed. As they emerge together from Γ point when *k*_*z*_ = 0, they will eventually cross each other along a closed loop with both slopes positive, forming a type-II nodal ring (Fig. 2b). We note that this red nodal ring is rather robust against choices of material and geometric parameters (see Supplementary Note 8). It is also an ideal type-II nodal ring without frequency variation because the plates without perforations are rotation-invariant. We have derived an effective Hamiltonian for the red nodal ring using the transfer matrix method (see Supplementary Note 9),

2 $${H}_{{{{{{\rm{eff}}}}}}}({{{{{\bf{k}}}}}})={v}_{r}({k}_{r}-{k}_{r0}){\sigma }_{3}+{v}_{z}{k}_{z}{\sigma }_{1}+{v}_{0}({k}_{r}-{k}_{r0}){\sigma }_{0},$$

where *k*_*r*0_ is the radius of the nodal ring, σ_*i*_ (*i* = 1, 2, 3) are Pauli matrices, σ_0_ is 2×2 identity matrix, and *v*_0_, *v*_*r*_, *v*_*z*_ are fitted velocity parameters, with |*v*_0_/*v*_*r*_ | > 1 manifested by the strong tilting of the two bands. On a closed loop encircling the type-II nodal ring, the bands obtained by solving the effective Hamiltonian of Eq. (2) agree excellently with those from full-wave simulations (Fig. 2c), further validating the analysis.

After a square lattice of perforations are introduced, the blue and green nodal rings emerge, while the red nodal ring is slightly distorted due to the breaking of rotation-invariance. Distinct from the robust red nodal rings associated with the plate thickness (see Supplementary Note 4), we note that the green and blue nodal rings are directly related to the through holes. To illustrate this point, instead of through holes, we also evaluate the case of blind holes on two sides of the metallic plates, with the limiting case that they perforate the metallic plates (see Supplementary Note 5). The evolution of the band structure reveals that only the red nodal ring, arising solely from the fluid-solid interaction (see Supplementary Note 16), can exist with blind holes (Fig. 2d). The green nodal rings could shrink (expand) in company with the expanding (shrinking) blue nodal rings as they are chained together^42^ due to the mirror symmetries *M*_*x*_ (*x* → −*x*) and *M*_*y*_ (*y* → −*y*). The blue nodal rings will reconnect if they touch each other when we tune geometric parameters and consequently the green nodal rings will be eliminated (see Supplementary Note 6). The blue and green nodal rings are largely owing to the acoustic resonance mode of the through holes (see Supplementary Note 17). Nevertheless, all the nodal rings are protected by corresponding mirror symmetries. They will all be gapped if there are no mirror symmetries in the phononic crystal (see Supplementary Note 7). Further, interface states of topological origin can be constructed by combining the phononic crystals that break the mirror symmetry in different ways^43^ (see Supplementary Note 18).

### Experimental observation of type-II nodal rings

**Fig. 3:**
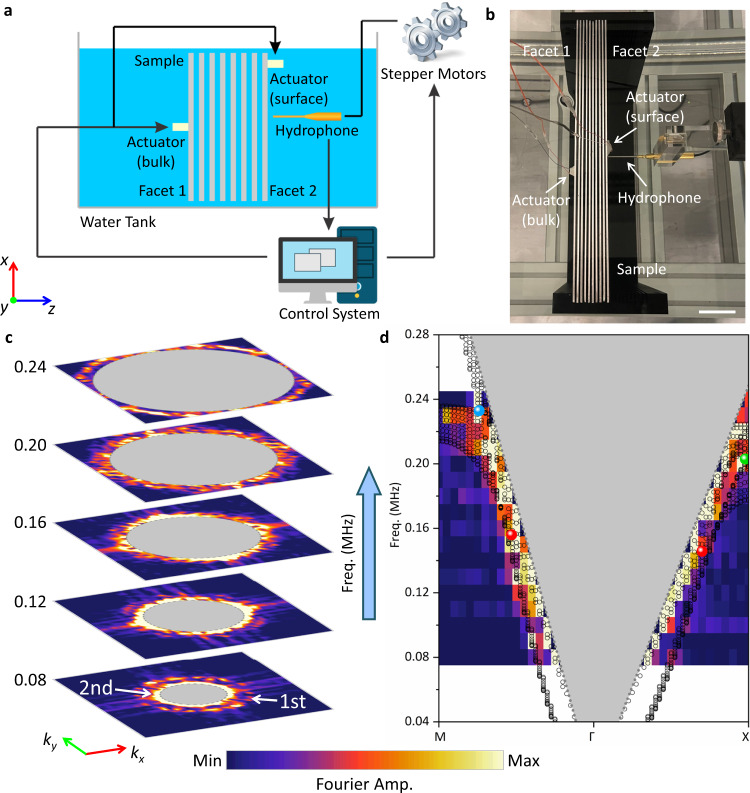
Experimental observation of type-II nodal rings. **a** Schematic of experimental setup for near-field scanning. A hydrophone is employed to image the ultrasound signal at facet 2. Piezoelectric actuators are used to actuate the phononic crystal. When detecting bulk bands, the actuator attached on facet 1 is excited, while detecting the surface states, the actuator attached on facet 2 is excited. **b** Photograph of the sample and experimental setup. White scale bar: 40 mm. **c** Spatial Fourier spectra of experimentally imaged fields at corresponding frequencies. The bright stripes correspond to modes of the 1st and 2nd bands excited and observed in experiments. Gray-shaded regions: the sound cone projected on the *k*_*x*_-*k*_*y*_ plane. **d** Experimental Fourier spectra along high-symmetry directions. Gray shaded region: the projected sound cone. Black circles: calculated band structure projected along *k*_*z*_. The bright stripes correspond to excited modes, which generally overlap with black circles from full-wave simulations. The colored dots represent the nodal rings of the same color denoted in Fig. 1.

Next, we perform ultrasonic near-field scanning in water to experimentally validate the type-II nodal rings^44^. When probing bulk bands with the experiment setup (Fig. 3a, see Methods for details), out-of-plane displacement, which couples efficiently with underwater ultrasound, is generated by a piezoelectric actuator. The actuator is attached on one side (*x-y* surface) of the sample, and the ultrasound field on the opposite side is detected point-by-point (Fig. 3b). The imaged fields are then Fourier transformed^14^ to extract the intensity spectra outside the sound cone in the reciprocal space (surface FBZ). The bright strips in the intensity spectra indicate excited bulk modes outside sound cone in experiments that is projected over *k*_*z*_, and their evolution with respect to frequency is presented (Fig. 3c). For better corroboration, we extract spatial Fourier spectra along high symmetry directions, and the occupied ranges of excited modes in surface FBZ also agree quite well with the calculated band structure projected over *k*_*z*_ (Fig. 3d). For further confirmation, we have carried out control experiments with in-plane lattice constant changed to *a*_0_ = 4 mm, and the results also agree with corresponding simulations (see Supplementary Note 20).

### Emergence of strongly tilted drumhead surface states

**Fig. 4:**
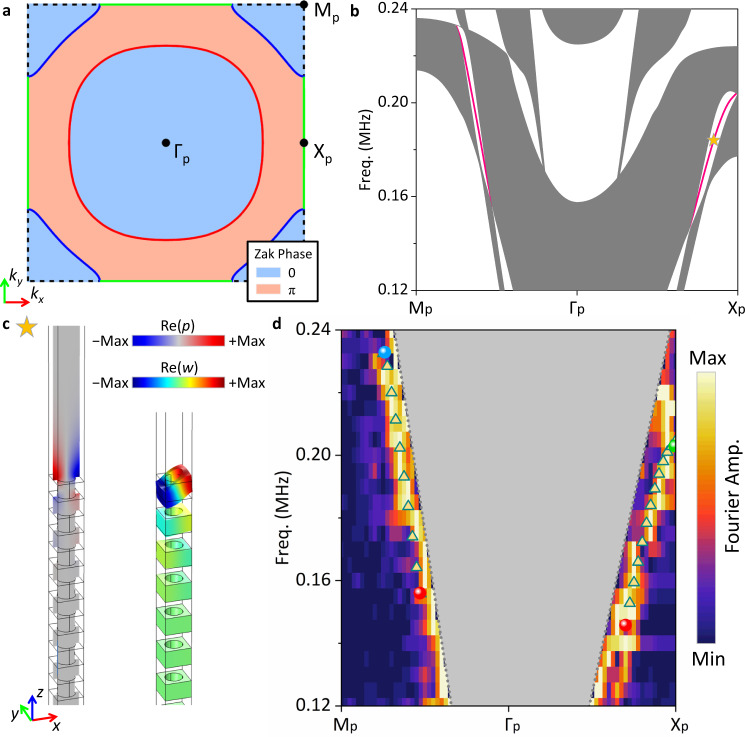
Strongly tilted drumhead surface state (DSS) between nodal rings. **a** Distribution of Zak phase on the surface FBZ projected along *z* direction. **b** Calculated projected band structure of a supercell terminated by *x*-*y* surface. Solid lines represent the strongly tilted DSS. Gray shaded regions denote the projected bulk bands. **c** Calculated field maps of acoustic pressure (Re(*p*)) and elastic displacements (Re(*u*), Re(*v*), Re(*w*)) for the marked point in (**b**). The thin solid lines outline profile of the supercell without elastic displacements. **d** Experimental Fourier spectra along high-symmetry directions when exciting and detecting the ultrasound at the same side of the phononic crystal. Triangle scatters: the calculated dispersion of the strongly tilted DSS along the high-symmetry directions.

To further elaborate the topological effects of type-II nodal rings, we also note the existence of drumhead surface state (DSS) between the red type-II nodal ring on *k*_*z*_ = 0 and the blue type-II nodal ring on *k*_*z*_ = ±π/*a*_z_. The DSS is closely related to the Zak phase along *k*_*z*_ direction that is quantized owing to the mirror symmetry *M*_*z*_ (*z* → −*z*) of the phononic crystal. The distribution of the Zak phase in the surface FBZ (Fig. 4a) is delineated by the projection of the two nodal rings on *k*_*x*_-*k*_*y*_ plane^45^ (see Supplementary Note 10 for numerical verifications), and it suggests possible existence of DSS in the region with Zak phase^12^ equal to π. The calculated projected band structure of a supercell terminated by its *x-y* surface confirms that a strongly tilted DSS emerges in the partial bandgap (Fig. 4b), a hallmark of the topological effect of the type-II nodal rings. This strong tilting is in sharp contrast to DSSs of type-I nodal rings, where they are generally flat. The calculated 3D dispersion of the DSS also demonstrates its strong tilting (see Supplementary Note 11). The field maps of the strongly tilted DSS (Fig. 4c), including both acoustic pressure and out-of-plane displacement, attests that the state is indeed localized on the surface of the phononic crystal, and is a hybridized mode with significant energy distributed in both water and plates (see Supplementary Note 12).

Finally, we seek to experimentally detect the strongly tilted DSS. Based on the essentially same experimental setup but the source and detector now on the same *x-y* side (Fig. 3(a)), we perform near-field scanning and the imaged fields (see Supplementary Note 13) are then Fourier transformed again. The excited modes revealed in the experimentally extracted Fourier spectra (Fig. 4d) reach good agreement with numerical calculations, thereby confirming the existence of the DSS along with its key signature, the strong tilting. Because we cannot prevent the excitation of bulk states, there are also bright stripes emerging at low-frequency range (see Supplementary Note 19). Control experiments are also carried out to further confirm our findings (see Supplementary Note 20).

## Discussion

In this work, we demonstrate an approach for topological phononics and with the approach, we realize type-II nodal rings in a phononic crystal, and experimentally capture the associated key topological phenomena with underwater ultrasound. The simple phononic crystal, only needing perforation during manufacturing, can be directly rescaled to higher frequency regimes. It may inspire on-chip devices with its propagating topological surface waves (strongly tilted DSSs). Generally, our approach reveals that for sound in fluids and solids, the interaction between their fundamentally different wave dynamics contains rich topological physics, with designs guided by the symmetry. For example, the type-II nodal rings can be gapped into ideal type-II Weyl points^13,35^ by lowering the symmetry of the phononic crystal (see Supplementary Note 14), and hence our findings expand topological systems and can serve as platforms to explore topological physics in a much simpler manner, not to mention the significantly reduced thermo-viscous losses of sound in water compared with air^46^. Remarkably, the rich physics discovered in this platform may also advance theoretical studies and experimental realizations of other nodal-line topologies, such as non-Abelian nodal links^31^ and topological charges^47^.

## Methods

### Simulations

In all simulations, the finite-element method software COMSOL Multiphysics is employed. The aluminum is modeled with density *ρ*_*m*_ = 2700 kg/m^3^, Young’s modulus *E* = 69 GPa, and Poisson’s ratio *ν* = 0.33, while the water is modeled with *ρ*_*w*_ = 1000 kg/m^3^ and speed of sound *c*_*w*_ = 1490 m/s. When calculating bulk band structures using a unit cell, periodic boundary conditions are applied on all boundaries. When calculating projected band structures using a supercell, periodic boundary conditions are applied on periodic boundaries and plane-wave radiation boundary conditions are applied on terminated boundaries to model finite structures. The terminated boundaries are five lattice constants away from finite structures, which proves to be appropriate as the modes of interest are evanescent along the *z* (out-of-plane) direction outside the structures. The maximum element size of the meshes in all simulations is 0.5 mm, which is ~1/10 of the wavelength of underwater ultrasound at 0.30 MHz.

### Experiments

The aluminum plates (pure aluminum) are perforated using a sheet metal punching machine and installed on a frame. Piezoelectric actuators (KEMET, USA) are attached on the outside facets of the aluminum plates as the source in experiments. A needle hydrophone mounted on a 3D motorized stage is used as the detector to collect the ultrasound data and perform near-field scanning. Signal of each frequency is emitted to excite the actuator, and corresponding time-domain signals are collected point-by-point using the hydrophone. The time-domain signals are Fourier transformed to obtain the field maps in the real space for each frequency. We then perform spatial Fourier transforms to retrieve band structures in the reciprocal space. The step resolution of scanning is 1 mm × 1 mm.

## Supplementary information

Supplementary Information

## Data Availability

The data which support the figures and other findings within this paper are available from the corresponding authors upon request.
