# Supplementary material for: Topological phononics arising from fluid-solid interactions
Source: Nat Commun. 2022 Oct 17;13:6120. doi: 10.1038/s41467-022-33896-4 (PMC9576708; doi:10.1038/s41467-022-33896-4)
Supplement: Supplementary file 1 — Supplementary Information [file 41467_2022_33896_MOESM1_ESM.pdf]

Supplementary Information

Topological phononics arising from fluid-solid interactions

Xiaoxiao Wu[1,2,a)], Haiyan Fan[3,a)], Tuo Liu[4], Zhongming Gu[5], Ruo-Yang Zhang[6], Jie Zhu[5,b)], Xiang Zhang[1,b)]

[1]*Faculties of Sciences and Engineering, The University of Hong Kong, Hong Kong, China*

[2]*Quantum Science and Technology Center and Advanced Materials Thrust, The Hong Kong University of Science and Technology (Guangzhou), Nansha, Guangzhou 511400, Guangdong, China*

[3]*Department of Mechanical Engineering, The Hong Kong Polytechnic University, Hung Hom, Kowloon, Hong Kong, China*

[4]*Key Laboratory of Noise and Vibration Research, Institute of Acoustics, Chinese Academy of Sciences, Beijing 100190, China*

[5]*Institute of Acoustics, School of Physics Science and Engineering, Tongji University, Shanghai 200092, China*

[6]*Department of Physics, The Hong Kong University of Science and Technology, Clear Water Bay, Kowloon, Hong Kong, China*

[a)]Xiaoxiao Wu and Haiyan Fan contributed equally to this work.

[b)]Correspondence and requests for materials should be addressed to J. Zhu (email: jiezhu@tongji.edu.cn), or X. Zhang (email: president@hku.hk).

Supplementary Note 1

Band structures slightly away from high-symmetry planes

To confirm the touching points between the 1st and 2nd bands are nodal rings, we calculate band structures along directions that are slightly away from high-symmetry planes. For comparison, the band structures on high-symmetry planes $k_z = 0$ and $k_z = \pi/a_z$ are plotted in Figs. S1(a) and S1(b). The calculated band structure with $k_z = 0.1\pi/a_z$ is shown in Fig. S1(c), which confirms that the red touching points in Fig. S1(a) are now gapped. We then consider the band structure with $k_z = 0.9\pi/a_z$, and the result in Fig. S1(d) confirms that the blue and green touching points in Fig. S1(b) are also gapped when slightly away from the high-symmetry planes.

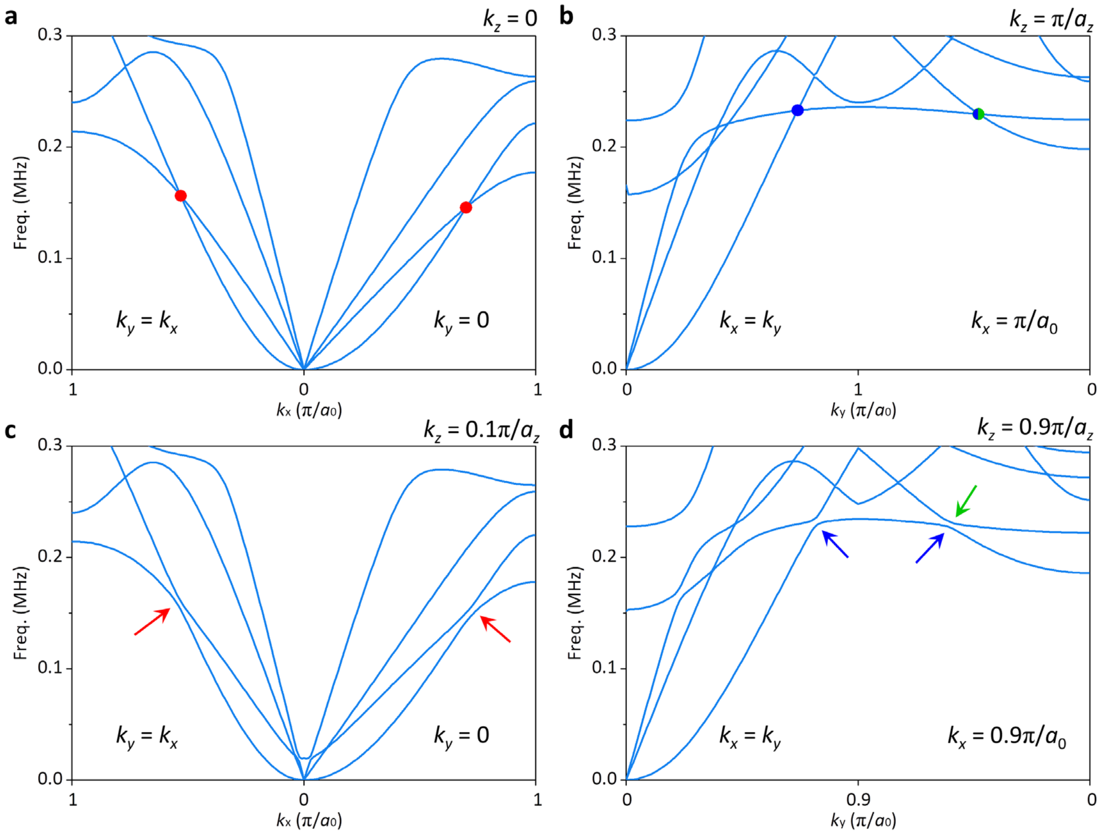

FIG. S1. Calculated band structures on planes slightly away from high-symmetry ones. (a)-(d) Calculated band structures on $k_z = 0$ (a), $k_z = \pi/a_z$ (b), $k_z = 0.1\pi/a_z$ (c) and

$k_z = 0.9\pi/a_z$ (d), respectively. The paths are indicated by equations in the figures, such as $k_y = k_x$ and $k_y = 0$. The touching points of the nodal rings demonstrated in (a) and (b), as denoted by the colored dots, are gapped correspondingly in (c) and (d), as indicated by the colored arrows.

Supplementary Note 2

Calculation of bands without fluid-solid interaction

To demonstrate that the fluid-solid interaction cannot be ignored in the “mixture” phononic crystal, we calculate the band structure without the interaction. In this case, the phononic crystal are modeled only with the Pressure Acoustics module in COMSOL Multiphysics. The longitudinal sound speed of aluminum $c_l = 6100$ m/s is employed in the numerical calculations, and the calculated band structure is shown in Fig. S2. It can be seen that only one mode, that is, the WG mode, emerges from Γ point. The SH mode and Lamb modes (both FL and EL modes) arising from solid displacements all disappear.

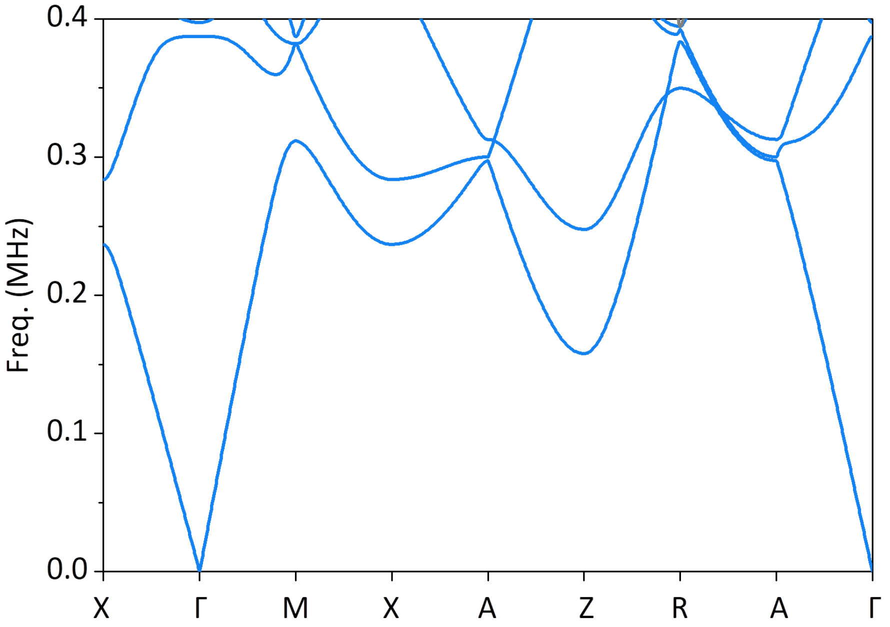

FIG. S2. Band structure of the phononic crystal without fluid-solid interaction. We only consider the longitudinal component of the system in the numerical calculations. Resultantly, the SH modes and Lamb modes (both FL and EL modes) that involve transverse components disappear.

Supplementary Note 3

Mode profiles of the first four bands around Γ point

We plot the mode profiles at $\mathbf{k} = (0.1\pi/a_0, 0, 0)$ for the modes of the first four bands in Fig. S3. It can be seen that when $k_z = 0$, the FL modes and WG modes have opposite parities with respect to the mirror symmetry M_z $(z \rightarrow -z)$.

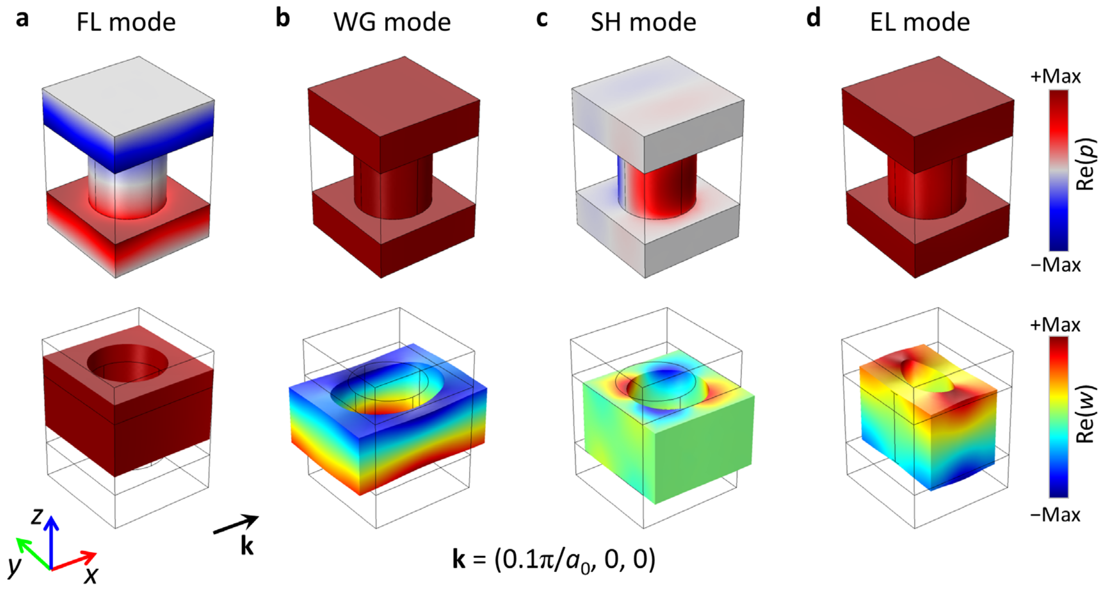

FIG. S3. Mode profiles of the first four bands around Γ point. (a)-(d) Calculated field maps of the modes at **k** = (0.1π/a_0, 0, 0), including the acoustic pressure (Re(p)) and elastic displacements (Re(u), Re(v), Re(w)). The thin solid lines outline the profile of the unit cells without elastic displacements.

Supplementary Note 4

Evolution of nodal rings when tuning thickness of plates

We consider the evolution of the nodal rings when we tune the thickness of plates t_{m}. The calculated band structures and distribution of nodal rings are demonstrated in Fig. S4. It can be seen that the red nodal ring is shrunken when we increase the thickness of plates t_m, as shown in Figs. S4(a) and S4(b). On the other hand, if we decrease the thickness of plates t_m, the red nodal ring will expand, touch each other, reconnect, and become centered around M point of FBZ, as shown in Fig. S4(c). With further expansion, the red nodal ring will disappear, as shown in Fig. S4(d).

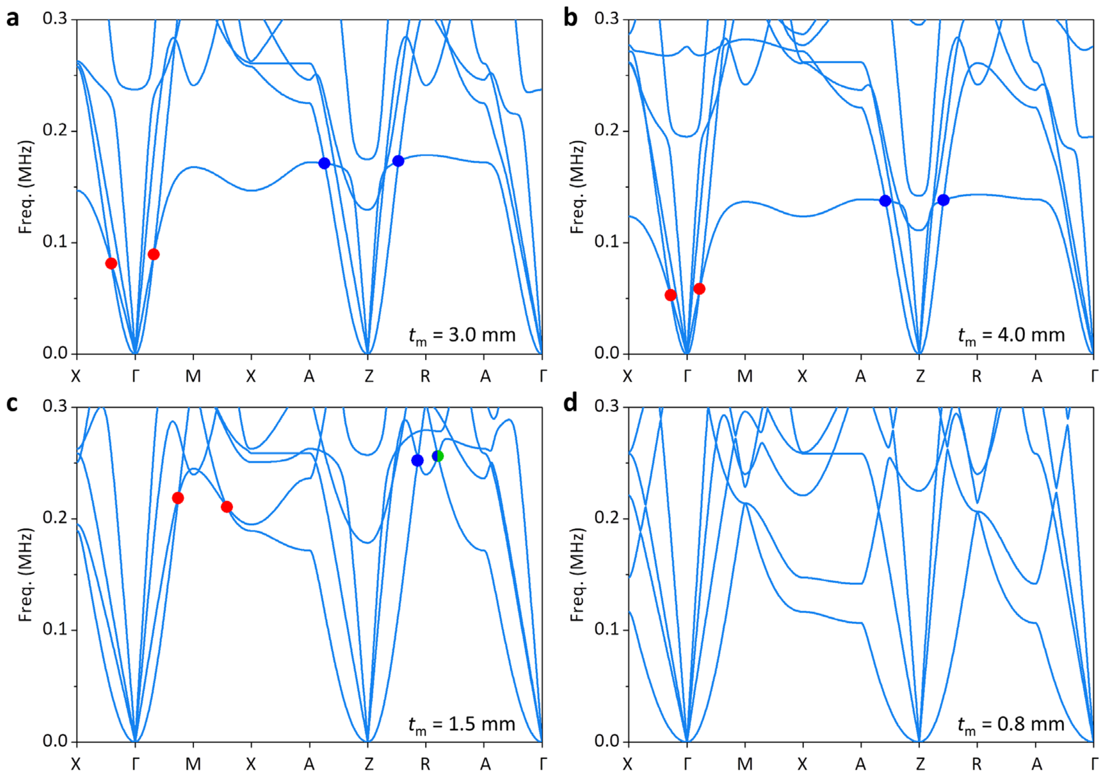

FIG. S4. Evolution of nodal rings when tuning thickness of plates. (a),(b) Calculated band structures when increasing thickness of plates, with t_m = 3.0 mm (a) and t_m = 4.0 mm (b), respectively. (c),(d) Calculated band structures when decreasing thickness of plates, with t_m = 1.5 mm (a) and t_m = 0.8 mm (b), respectively.

Supplementary Note 5

Emergence of blue and green nodal rings

To illuminate the origin of blue and green nodal rings, we start from metallic plates without holes as considered in Fig. 2(a). We introduce a lattice of circular blind holes instead of through holes on the metallic plates, with the thickness of the middle layer being t_h. The calculated band structure in Figs. S5(a) and S5(b) show that only the red nodal ring exists even when t_h = 0.4 mm. In comparison, we introduce a square lattice of circular through holes with diameter d_0 = 0.4 mm and d_0 = 1.2 mm,

respectively. The corresponding band structures are displayed in Figs. S5(c) and S5(d). The green and blue nodal rings exist for these through holes, and the diameter of holes d_0 only slightly affect them, which suggest their emergence are highly dependent on the acoustic resonance of the through holes. For more discussion, please see Supplementary Note 17.

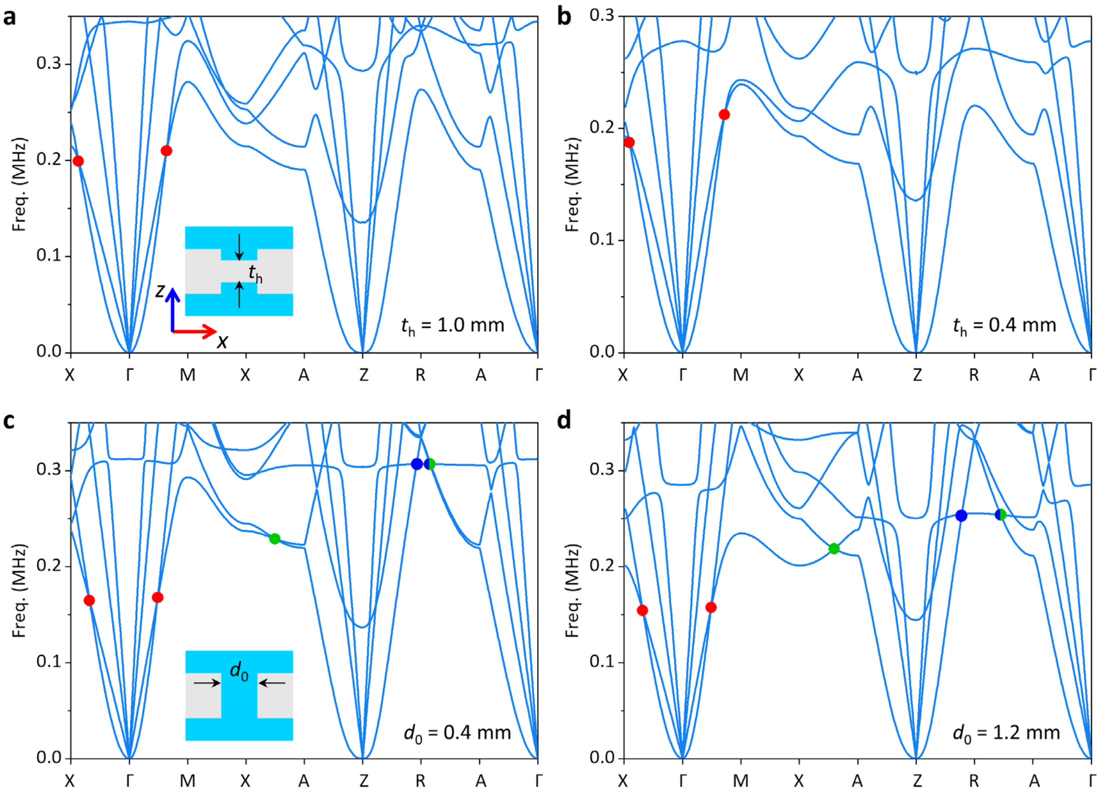

FIG. S5. Emergence of blue and green nodal rings. (a),(b) Calculated band structures when the holes are blind holes, with t_h = 1.0 mm and t_h = 0.4 mm, respectively. (c),(d) Calculated band structures when the holes are through holes, with d_0 = 0.4 mm and d_0 = 1.2 mm, respectively. Insets: sectional schematics of the unit cells.

Supplementary Note 6

Evolution of nodal rings when tuning separation of plates

We consider the evolution of the nodal rings when we tune the separation of plates t_w. The calculated band structures are shown in Figs. S6(a)-S6(c). It can be seen that the blue nodal rings expand in company with the green nodal ring shrinking when we enlarge separation of plates t_w. When the blue nodal rings touch each other, they will reconnect, centered around the Z point instead of the R point of the FBZ, and the green nodal rings are eliminated, as demonstrated in Figs. S6(d)

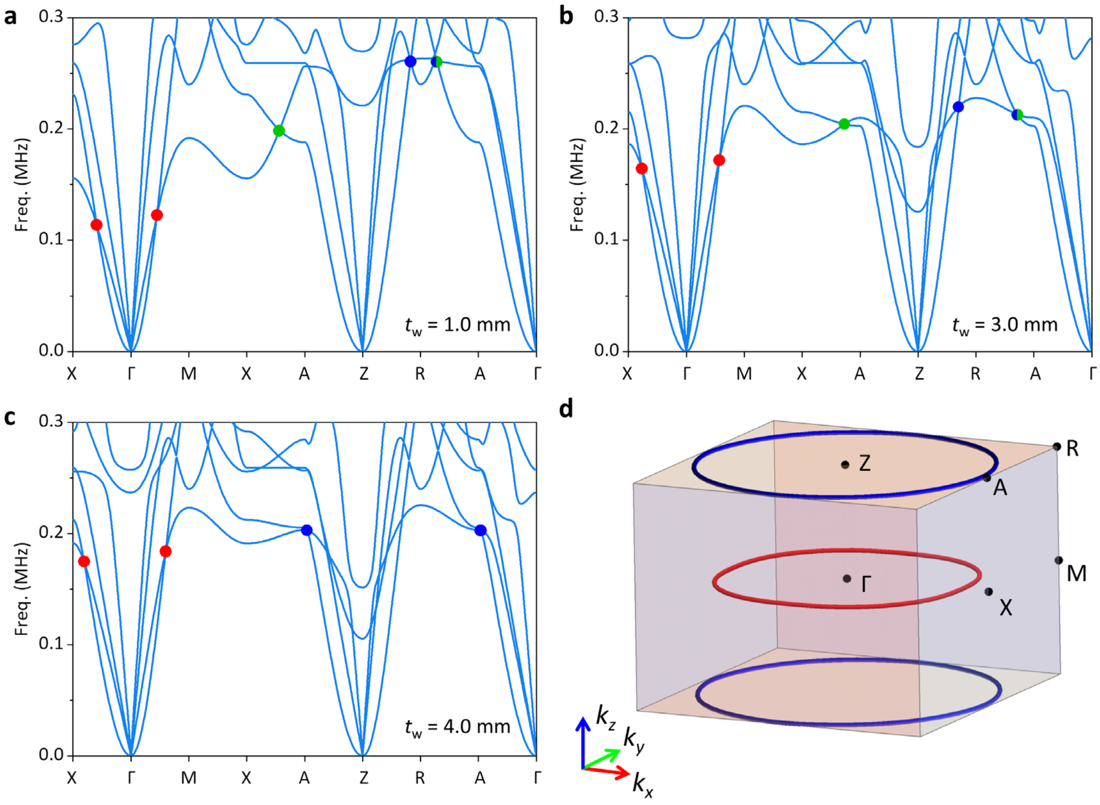

FIG. S6. Evolution of nodal rings when tuning separation of plates. (a)-(c) Calculated band structures when t_w = 1.0 mm (a), t_w = 3.0 mm (b), and t_w = 4.0 mm (c). (d) Distribution of the nodal rings for t_w = 4.0 mm (c).

Supplementary Note 7

Band structure with no mirror symmetries

We consider a unit cell with no mirror symmetries as shown in Fig. S7(a). It consists of two holes with different diameters on the two sides of the aluminum plate, which breaks the mirror symmetry M_z ($z \to -z$). To break the other mirror symmetries M_x ($x \to -x$) and M_y ($y \to -y$), we also shift the holes on the back side of the aluminum plate with the displacement on the x (y) direction being δ_x (δ_y), as indicated in the inset of Fig. S7(a). The detailed geometric parameters we choose are d_1 = 2.4 mm, d_2 = 0.8 mm, t_1 = 1.0 mm, t_2 = 1.0 mm, δ_x = 0.5 mm, and δ_y = 0.5 mm. The calculated band structure along high-symmetry directions is shown in Fig. S7(b), and it can be seen that all touching points between the 1st and the 2nd bands are gapped now, as detailedly demonstrated in Figs. S7(c) and S7(d).

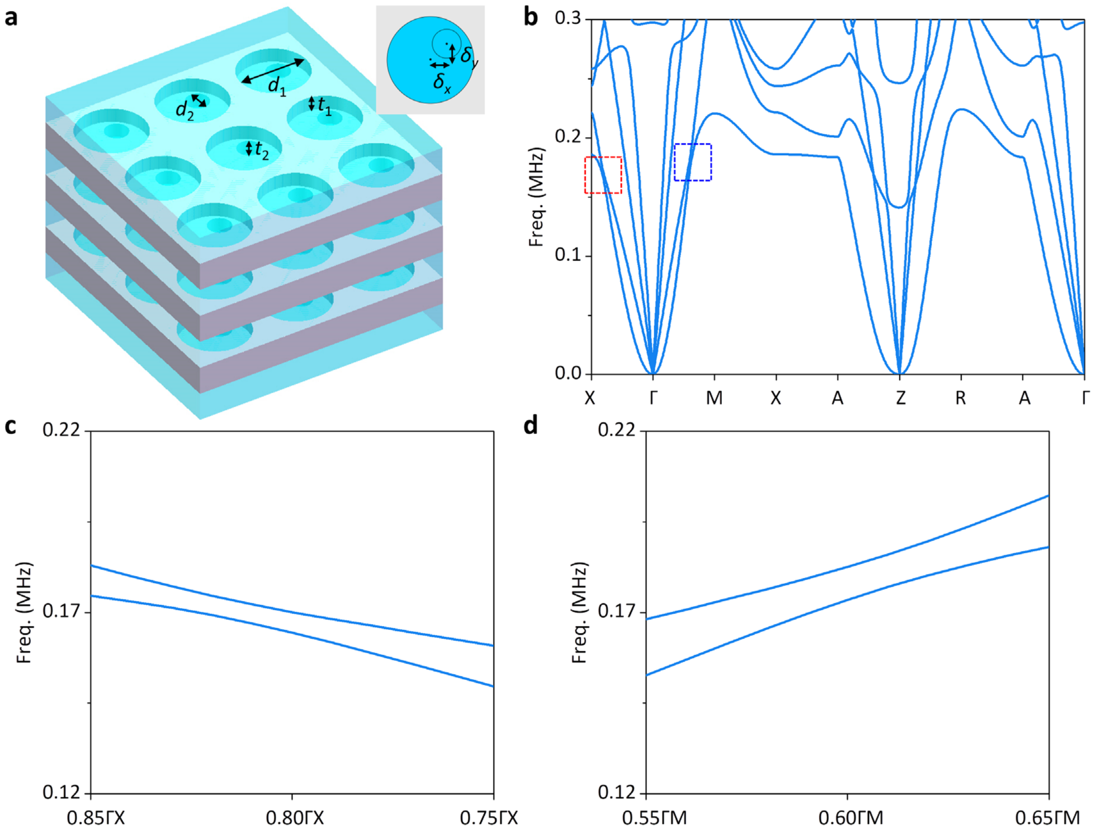

FIG. S7. Band structure of the phononic crystal with no mirror symmetries. (a) Perspective schematic of the phononic crystal with no mirror symmetries. Inset: the

holes on the back side of the aluminum plate are shifted. (b) The calculated band structure along high-symmetry directions of the phononic crystal. (c),(d) Enlarged views for the regions denoted by the red (c) and blue (d) dashed boxes in (b), respectively.

Supplementary Note 8

Comparison with the phononic crystal immersed in air

We calculate the band structure of the phononic crystal immersed in air, and it can be seen that the deterministic type-II nodal ring on $k_z = 0$ plane still exists, as shown in Fig. S8(a). However, it is obvious that the size of the nodal ring is significantly shrunken due to the smaller sound speed of air compared with water. Further, the sound is also much more difficult to be transmitted due to the extreme impedance mismatch between air and aluminum which leads to very weak fluid-solid interaction. In fact, we have simulated excitation of the phononic crystal in water and air at the corresponding frequency of the red nodal ring on $k_z = 0$, respectively. In simulations, the phononic crystal is comprised of 10 layers of the drilled aluminum plates, and the average out-of-plane displacement for each layer is shown in Fig. S8(b). It is seen that the sound can hardly be transmitted when the phononic crystal is immersed in air, since the specific acoustic impedance of aluminum is four orders of magnitude larger than that of air.

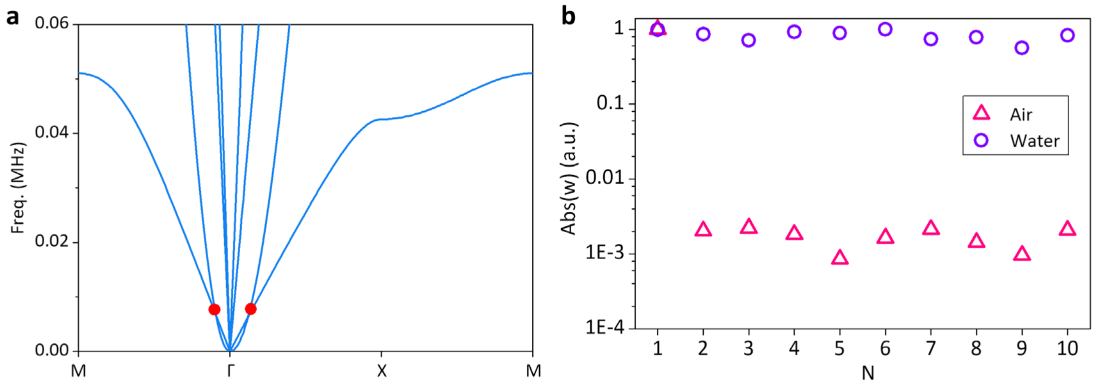

FIG. S8. Comparison with the phononic crystal immersed in air. (a) The calculated band structure with k_z = 0 when the phononic crystal is immersed in air. The red dots represent the nodal ring on k_z = 0. (b) The normalized amplitude of averaged out-of-plane displacement (Abs(w)) for each layer of the aluminum plates when excited at the frequency of the nodal ring on k_z = 0 for air and water, respectively. The excitation source is placed at the surface (N = 1).

Supplementary Note 9

Calculation of dispersions and derivation of effective Hamiltonian

Here, we use a transfer matrix method to model the phononic crystal that is comprised of periodic solid plates (aluminum) without holes immersed in the background fluid (water). To obtain an analytical model, we first calculate the dispersions of the first two modes of the phononic crystal in the long-wavelength limit, which allows us to model the solid plates based on the thin plate theory. We assume the time-harmonic condition and follow the $e^{-i\omega t}$ sign convention. As shown in Fig. S9(a), we assume k_y = 0 for simplicity as the system is rotation-invariant, and the acoustic pressure in the n-th water region can be written as

$$p_n(x,z) = A_n e^{ik_x x} e^{i\kappa_z z} + B_n e^{ik_x x} e^{-i\kappa_z z}, \tag{S1}$$

in which $\kappa_z = \sqrt{\omega^2 / c_w^2 - k_x^2}$ is the wave number of acoustic wave in z direction, while A_n and B_n are complex amplitudes of the forward and backward going acoustic waves, respectively. Likewise, the acoustic pressure in the (n+1)-th water region is then

$$p_{n+1}(x,z) = A_{n+1} e^{ik_x x} e^{ik_z (z-a_z)} + B_{n+1} e^{ik_x x} e^{-ik_z (z-a_z)}. \tag{S2}$$

The out-of-plane displacement in the n-th solid plate can be written as

$$w_n(x) = C_n e^{ik_x x}. \tag{S3}$$

The thin plate theory then gives the dynamic equation for the n-th solid plate

$$D\nabla_r^4 w(x) - p_n(x,z)\big|_{z=t_w} + p_{n+1}(x,z)\big|_{z=a_z} = \rho_m t_m \omega^2 w(x), \tag{S4}$$

where $D = \dfrac{Et_m^3}{12(1-\nu^2)}$ is the bending stiffness of the thin plate, and $\nabla_r = (\partial_x, \partial_y)$ is the gradient on the plate surface. From Eq. (S4), we can solve C_n as the function of A_n, B_n, A_{n+1}, and B_{n+1}

$$C_n = \frac{A_n e^{i\kappa_z t_w} + B_n e^{-i\kappa_z t_w} - A_{n+1} - B_{n+1}}{Dk_x^4 - \omega^2 \rho_m t_m}. \tag{S5}$$

Then, we use the boundary conditions of continuous velocity on two surfaces of the n-th solid plate, which give

$$\begin{aligned} \left.\frac{\partial w_n(x)}{\partial t}\right|_{z=t_w} &= v_{z,n}(x,z)\big|_{z=t_w} \\ \left.\frac{\partial w_n(x)}{\partial t}\right|_{z=a_z} &= v_{z,n+1}(x,z)\big|_{z=a_z} \end{aligned}, \tag{S6}$$

and it can be simplified under the time-harmonic condition as

$$
\begin{aligned}
-i\omega w_n(x)\big|_{z=t_w} &= \frac{1}{i\omega\rho_w}\frac{\partial p_n(x,z)}{\partial z}\bigg|_{z=t_w} \\
-i\omega w_n(x)\big|_{z=a_z} &= \frac{1}{i\omega\rho_w}\frac{\partial p_{n+1}(x,z)}{\partial z}\bigg|_{z=a_z}
\end{aligned}
\quad . \tag{S7}
$$

Combining Eqs. (S5) and (S7), we can eliminate C_n and solve A_{n+1} and B_{n+1} as the function of A_n and B_n, and we obtain the transfer matrix M

$$
\begin{bmatrix} A_{n+1} \\ B_{n+1} \end{bmatrix} = M \begin{bmatrix} A_n \\ B_n \end{bmatrix} = \begin{bmatrix} M_{11} & M_{12} \\ M_{21} & M_{22} \end{bmatrix} \begin{bmatrix} A_n \\ B_n \end{bmatrix}, \tag{S8}
$$

with the matrix element

$$
\begin{aligned}
M_{11} = M_{22}^* &= \frac{1}{2\omega^2\rho_w} e^{i\kappa_z t_w}[-ik_x^4\kappa_z D + \omega^2(2\rho_w + i\kappa_z\rho_m t_m)] \\
M_{12} = M_{21}^* &= \frac{1}{2\omega^2\rho_w} i e^{-i\kappa_z t_w}\kappa_z(k_x^4 D - \omega^2\rho_m t_m)
\end{aligned}
\quad , \tag{S9}
$$

where * denotes the complex conjugate. Since the system is periodic and Hermitian, we can utilize the criteria on the trace of the transfer matrix which states that [1]

$$
\mathrm{tr}(M) = 2\cos(k_z a_z), \tag{S10}
$$

where k_z is the Bloch wave number in z direction. Then, it gives the equation which determines the dispersions of the modes

$$
\frac{2\omega^2\rho_m\cos(\kappa_z t_w) + \kappa_z(Dk_x^4 - \omega^2\rho_m t_m)\sin(\kappa_z t_w)}{\omega^2\rho_w} = 2\cos(k_z a_z)\,. \tag{S11}
$$

When $k_z = 0$, one solution gives the WG mode,

$$
\omega_{\mathrm{WG}} = k_x c_w, \tag{S12}
$$

which coincides with the sound cone projected over k_z. The other solution in the long-wavelength limit when $k_z = 0$ gives the FL mode,

$$
\omega_{\mathrm{FL}} = k_x^2\sqrt{\frac{D}{\rho_m t_m + \rho_w t_w}}\,. \tag{S13}
$$

As can be seen, this dispersion is similar to that of a thin plate in free space $\omega = k_x^2 \sqrt{D/(\rho_m t_m)}$, except its quadratic coefficient is reduced due to the fluid-solid interaction. We then plot the first two bands around Γ point from full-wave simulations and from Eqs. (S12) and (S13) in Fig. S9(b), and they agree quite well.

We then derive the effective Hamiltonian of the red nodal ring. Equating the two expressions $\omega_{\mathrm{WG}} = \omega_{\mathrm{FL}}$, it is found that the two modes will cross each other at

$$k_{x0} = c\sqrt{\frac{\rho_m t_m + \rho_w t_w}{D}}. \tag{S14}$$

Based on Eq. (S14), we plot the evolution of k_{x0} with respect to t_{w} and compare that with values extracted from full-wave simulations, and the results agree quite well. Next, we expand Eq. (S11) around $\mathbf{k}_0 = (k_{x0}, 0, 0)$ and $\omega_0 = ck_{x0}$ to the second order of δk_x, k_z, and $\delta\omega$, and we obtain the equation

$$\begin{aligned}&t_w(\rho_m t_m + \rho_w t_w)(12D + c^2\rho_w t_w^3)\delta\omega^2\\&-2ct_w(\rho_m t_m + \rho_w t_w)(18D + c^2\rho_w t_w^3)\delta\omega\delta k_x\\&+c^2 t_w(\rho_m t_m + \rho_w t_w)(24D + c^2\rho_w t_w^3)\delta k_x^2 + 3(t_m + t_w)^2 c^2 \rho_w D k_z^2 = 0\end{aligned}, \tag{S15}$$

where $\delta k_x = k_x - k_{x0}$ and $\delta\omega = \omega - \omega_0$. Comparing Eq. (S15) with the characteristic equation of the effective Hamiltonian

$$H_{\mathrm{eff}} = \begin{bmatrix} v_0\delta k_x + v_r\delta k_x & v_z k_z \\ v_z k_z & v_0\delta k_x - v_r\delta k_x \end{bmatrix}, \tag{S16}$$

which is

$$\det(H_{\mathrm{eff}} - \delta\omega I) = \delta\omega^2 - 2v_0\delta k_x\delta\omega + v_0^2\delta k_x^2 - v_x^2\delta k_x^2 - v_z^2\delta k_z^2, \tag{S17}$$

we can conclude that the parameters are

$$\begin{aligned} v_0 &= c_w + \frac{6Dc_w}{12D + c_w^2 \rho_w t_w^3} \\ v_r &= \frac{6Dc_w}{12D + c_w^2 \rho_w t_w^3} \\ v_z &= c(t_m + t_w)\sqrt{\frac{3\rho_w D}{t_w(\rho_m t_m + \rho_w t_w)(12D + c^2 \rho_w t_w^3)}} \end{aligned}, \tag{S18}$$

and obviously we have $|v_0/v_r|>1$, confirming the touching points form a type-II nodal ring. We need to point out that, since k_{x0} is not small enough for the thin plate assumption regarding our geometric parameters, the derivation above based on the thin plate assumption overestimate the slope of the FL mode v_0+v_x, and also underestimate the value of k_{x0}, as shown in Fig. S9(c). In contrast, the expression for v_z can quantitatively predict the dispersions along k_z direction since we start from $k_z = 0$, as shown in Fig. S9(d). The effective Hamiltonian in the main text can be obtained after we replace the x direction with a general direction in x-y plane, that is, k_x is replaced by k_r, k_{x0} is replaced by k_{r0}, etc.

To correct the values of k_{x0}, we have also considered a rigorous model of full elasticity instead of the thin plate theory. With full elasticity, the in-plane displacement u_n and out-of-plane displacement w_n are expressed using the scalar potential $\varphi_n(x, z)$ and the vector potential (z component) $\psi_n(x, z)$

$$\begin{aligned} u_n(x,z) &= \frac{\partial \varphi_n}{\partial x} - \frac{\partial \psi_n}{\partial z} \\ w_n(x,z) &= \frac{\partial \varphi_n}{\partial z} + \frac{\partial \psi_n}{\partial x} \end{aligned}. \tag{S19}$$

The potentials are expressed as

$$\begin{aligned} \varphi_n(x,z) &= C_{1n} e^{ik_x x} \cosh(\kappa_{\mathrm{d}} z) + C_{2n} e^{ik_x x} \sinh(\kappa_{\mathrm{d}} z) \\ \psi_n(x,z) &= D_{1n} e^{ik_x x} \cosh(\kappa_{\mathrm{s}} z) + D_{2n} e^{ik_x x} \sinh(\kappa_{\mathrm{s}} z) \end{aligned}, \tag{S20}$$

where $\kappa_{\mathrm{d}} = \sqrt{k_x^2 - \omega^2 / c_l^2}$ and $\kappa_s = \sqrt{k_x^2 - \omega^2 / c_{\mathrm{s}}^2}$, with c_l being the longitudinal

sound velocity and c_s being the shear sound velocity of the aluminum, respectively. Likewise, the acoustic pressure and velocities in water are expressed with the velocity potential $\phi_n(x, z)$

$$\begin{aligned} p_n &= i\omega\rho_w\phi_n \\ v_{x,n} &= \frac{\partial\phi_n}{\partial x} \\ v_{z,n} &= \frac{\partial\phi_n}{\partial z} \end{aligned} , \tag{S21}$$

and the velocity potential is expressed as

$$\phi_n(x,z) = A_n e^{ik_x x} e^{i\kappa_z z} + B_n e^{ik_x x} e^{-i\kappa_z z} , \tag{S22}$$

with $\kappa_z = \sqrt{\omega^2 / c^2 - k_x^2}$. We then can derive the transfer matrix of $[A_n, B_n]^T$ using the six boundary conditions [2], that is, the continuity of normal velocity on two surfaces of the n-th solid plate

$$\begin{aligned} \left.\frac{\partial w_n(x)}{\partial t}\right|_{z=t_w} &= \left. v_{z,n}(x,z)\right|_{z=t_w} \\ \left.\frac{\partial w_n(x)}{\partial t}\right|_{z=a_z} &= \left. v_{z,n+1}(x,z)\right|_{z=a_z} \end{aligned} , \tag{S23}$$

and the continuity of stress on the two surfaces

$$\begin{aligned} \left.\sigma_{zz,n}(x,z)\right|_{z=t_w} &= -\left. p_{z,n}(x,z)\right|_{z=t_w} \\ \left.\sigma_{zz,n}(x,z)\right|_{z=a_z} &= -\left. p_{z,n+1}(x,z)\right|_{z=a_z} \\ \left.\sigma_{xz,n}(x,z)\right|_{z=t_w} &= 0 \\ \left.\sigma_{xz,n}(x,z)\right|_{z=a_z} &= 0 \end{aligned} . \tag{S24}$$

The normal stress and shear stress are calculated as

$$\begin{aligned} \sigma_{xz,n}(x,z) &= \frac{E}{2(1+\nu)}\left(\frac{\partial u_n}{\partial z} + \frac{\partial w_n}{\partial x}\right) \\ \sigma_{zz,n}(x,z) &= \frac{E\nu}{(1+\nu)(1-2\nu)}\frac{\partial u_n}{\partial x} + \frac{E(1-\nu)}{(1+\nu)(1-2\nu)}\frac{\partial w_n}{\partial z} \end{aligned} . \tag{S25}$$

After elimination of C_{1n}, C_{2n}, D_{1n}, and D_{2n} by combining Eqs. (S23)-(S25), we obtain

the transfer matrix M_{full}

$$\begin{bmatrix} A_{n+1} \\ B_{n+1} \end{bmatrix} = M_{\text{full}} \begin{bmatrix} A_n \\ B_n \end{bmatrix}, \tag{S26}$$

from which we can numerically retrieve the k_{x0}. In this semi-analytical way, the retrieved k_{x0} from the full-elasticity theory are also plotted in Fig. S9(c), and they agree excellently with those from full-wave simulations. Therefore, it confirms that the error in our model is indeed due to the thin plate assumption. Nevertheless, the analytical model validates that the deterministic type-II nodal ring originates from the interplay of ultrasound in the aluminum plates and water, which results in different asymptotic behaviors of the FL and WG modes in the long-wavelength limit.

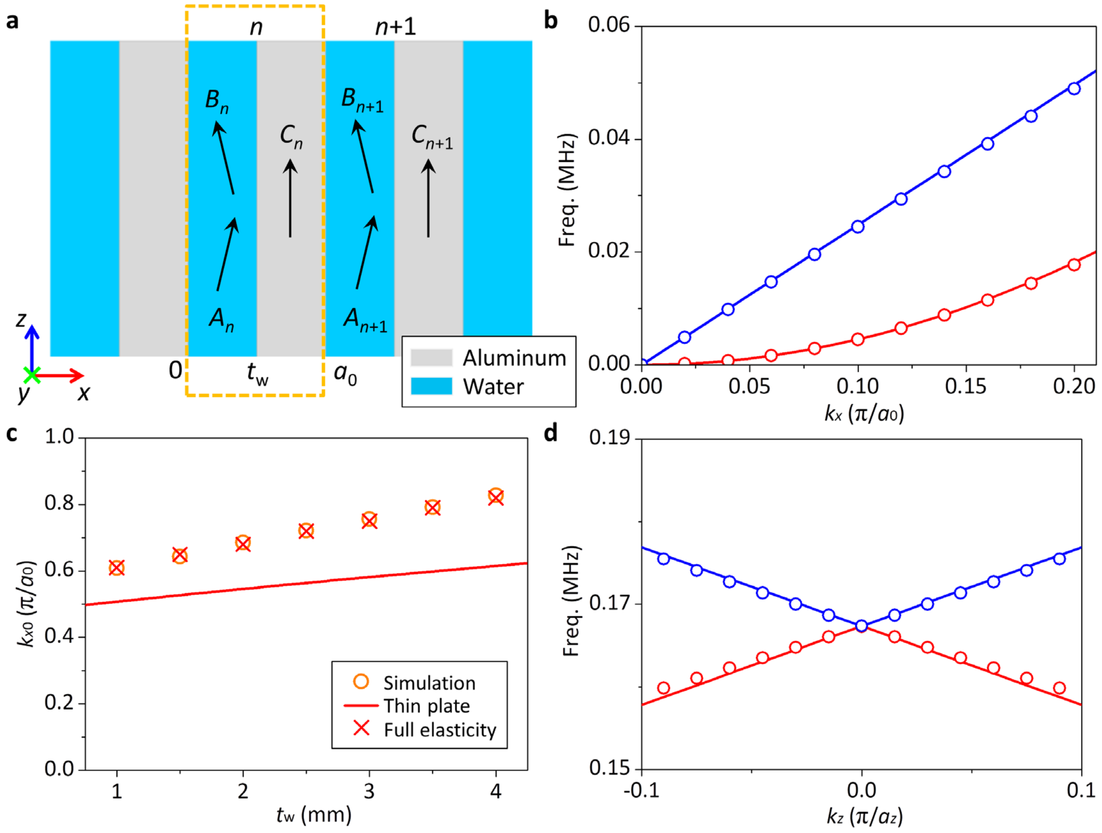

FIG. S9. Calculation of dispersions and derivation of effective Hamiltonian. (a) Schematic of the setup used to calculate dispersions with the transfer matrix method.

(b) First two bands around Γ point calculated from full-wave simulations (solid lines) compared with the dispersions based on the thin plate theory (dots). (c) Evolution of k_{x0} with respect to the separation of plates t_w. The solid line is calculated from the thin plate theory. The dots and crosses are retrieved from full-wave simulations and the full-elasticity theory, respectively. (d) Dispersions around the nodal ring along k_z direction calculated from full-wave simulations (solid lines) compared with dispersions derived from the thin plate theory (dots).

Supplementary Note 10

Numerical calculation of Zak phases

An eigenmode of the phononic crystal which includes fluid-solid interaction is generally comprised of ultrasound in both the fluid domains (described by acoustic pressure p) and the solid domains (described by displacement $\mathbf{u}$). We can define the inner product of two eigenmodes $|n_1\rangle$ and $|n_2\rangle$ of the phononic crystal as [3,4]

$$\begin{aligned}\langle n_1|n_2\rangle = &\int_{V_{\mathrm{w}}} \frac{1}{4\rho_{\mathrm{w}} c_{\mathrm{w}}^2} p_1^*(\mathbf{r}) p_2(\mathbf{r}) \mathrm{d}^3\mathbf{r} + \int_{V_{\mathrm{w}}} \frac{\rho_{\mathrm{w}}}{4} \mathbf{v}_{\mathrm{w}1}^*(\mathbf{r}) \cdot \mathbf{v}_{\mathrm{w}2}(\mathbf{r}) \mathrm{d}^3\mathbf{r} \\ &+ \int_{V_{\mathrm{m}}} \frac{1}{4} \mathbf{e}_1^*(\mathbf{r}) \cdot \mathbf{C} \cdot \mathbf{e}_2(\mathbf{r}) \mathrm{d}^3\mathbf{r} + \int_{V_{\mathrm{m}}} \frac{\rho_{\mathrm{m}}}{4} \mathbf{v}_{\mathrm{m}1}^*(\mathbf{r}) \cdot \mathbf{v}_{\mathrm{m}2}(\mathbf{r}) \mathrm{d}^3\mathbf{r}\end{aligned}, \quad \text{(S27)}$$

where V_{w} and V_{m} denoting the fluid and solid domains of the unit cell, respectively. In the integrals, $p_1(\mathbf{r})$ and $p_2(\mathbf{r})$ are periodic part of pressure field for $|n_1\rangle$ and $|n_2\rangle$, $\mathbf{e}_1(\mathbf{r})$ and $\mathbf{e}_2(\mathbf{r})$ are periodic part of strain field for $|n_1\rangle$ and $|n_2\rangle$. The elastic strain tensor $\mathbf{e}$ is defined as

$$e_{ij} = \frac{1}{2}\left(\frac{\partial u_i}{\partial x_j} + \frac{\partial u_j}{\partial x_i}\right), \quad \text{(S28)}$$

where u_i (i=1,2,3) are components of displacement and x_i (i=1,2,3) are coordinates, respectively. **C** is the stiffness tensor connecting stress and strain of the solid, which is expressed as

$$\begin{bmatrix} \sigma_{11} \\ \sigma_{22} \\ \sigma_{33} \\ \sigma_{12} \\ \sigma_{13} \\ \sigma_{23} \end{bmatrix} = \mathbf{C} \begin{bmatrix} e_{11} \\ e_{22} \\ e_{33} \\ 2e_{12} \\ 2e_{13} \\ 2e_{23} \end{bmatrix}, \tag{S29}$$

with

$$\mathbf{C} = \frac{E}{(1+\nu)(1-2\nu)} \begin{bmatrix} 1-\nu & \nu & \nu & & & \\ \nu & 1-\nu & \nu & & & \\ \nu & \nu & 1-\nu & & & \\ & & & \frac{1}{2}-\nu & & \\ & & & & \frac{1}{2}-\nu & \\ & & & & & \frac{1}{2}-\nu \end{bmatrix}. \tag{S30}$$

For simplicity, the symmetric 3×3 strain tensor is rearranged into a 6-component vector $\mathbf{e} = [e_{11}, e_{22}, e_{33}, 2e_{12}, 2e_{13}, 2e_{23}]^{\mathrm{T}}$. The particle velocity of ultrasound in the fluid is

$$\mathbf{v}_{\mathrm{w}} = \frac{\nabla p}{i\omega\rho_{\mathrm{w}}}, \tag{S31}$$

while the particle velocity of ultrasound in the solid is

$$\mathbf{v}_{\mathrm{m}} = -i\omega\mathbf{u}. \tag{S32}$$

Then, the eigenmodes can be normalized, and the Berry connection of the l-th band is then

$$B_l(\mathbf{k}) = i\langle n_{l,\mathbf{k}} | \nabla_{\mathbf{k}} n_{l,\mathbf{k}} \rangle, \tag{S33}$$

where $\left|n_{l,\mathbf{k}}\right\rangle$ is the normalized eigenstate of l-th band at $\mathbf{k}$ in the reciprocal space. Correspondingly, the Zak phase $\theta_l^{\text{Zak}}(k_x,k_y)$ along k_z direction of l-th band can be directly evaluated as (up to 2π)

$$\theta_l^{\text{Zak}}(k_x,k_y)=\int_{-\pi/a_z}^{\pi/a_z} B_{l,z}(\mathbf{k})\mathrm{d}k_z\ . \tag{S34}$$

In our numerical calculations, we discretize Eq. (S34), and the results for the 1st band of selected lines in the surface FBZ are plotted in Fig. S10 for comparison and verification. It can be seen that the numerical results agree quite well with the theoretical results inferred from the distribution of nodal rings. The numerical errors are within ±0.05, and can be further decreased by refining the discretization of k_z.

In fact, since the phononic crystal has mirror symmetry with respect to z direction, the Zak phase θ_1^{Zak} can also be inferred from parities of the eigenmodes at high symmetry points along the k_z path (k_z = 0 and π/a_z). Namely, we have [4]

$$\frac{\theta_1^{\text{Zak}}}{\pi}=\{\frac{1}{2}[M_{z,1}(k_z=0)-M_{z,1}(k_z=\pi/a_z)]\}\bmod 2, \tag{S35}$$

where $M_{z,1}$ are parities (±1) for mirror symmetry M_z ($z \rightarrow -z$) of the 1st band at k_z = 0 and π/a_z, respectively. The field maps of the eigenmodes at k_z = 0 and $k_z = \pi/a_z$ for $\mathbf{k}_r$ = (k_x, k_y) = ($0.6\pi/a_0$, 0) and ($0.8\pi/a_0$, 0) are shown in Figs. S10(c) and S10(d), respectively. It shows that, for mirror symmetry M_z, the eigenmodes at k_z = 0 and π/a_z have the same parity when $\mathbf{k}_r$ = ($0.6\pi/a_0$, 0), but opposite parities when $\mathbf{k}_r$ = ($0.8\pi/a_0$, 0). This contrast confirms that the Zak phase takes the value 0 for the former case and π for the latter case, as we have numerically demonstrated.

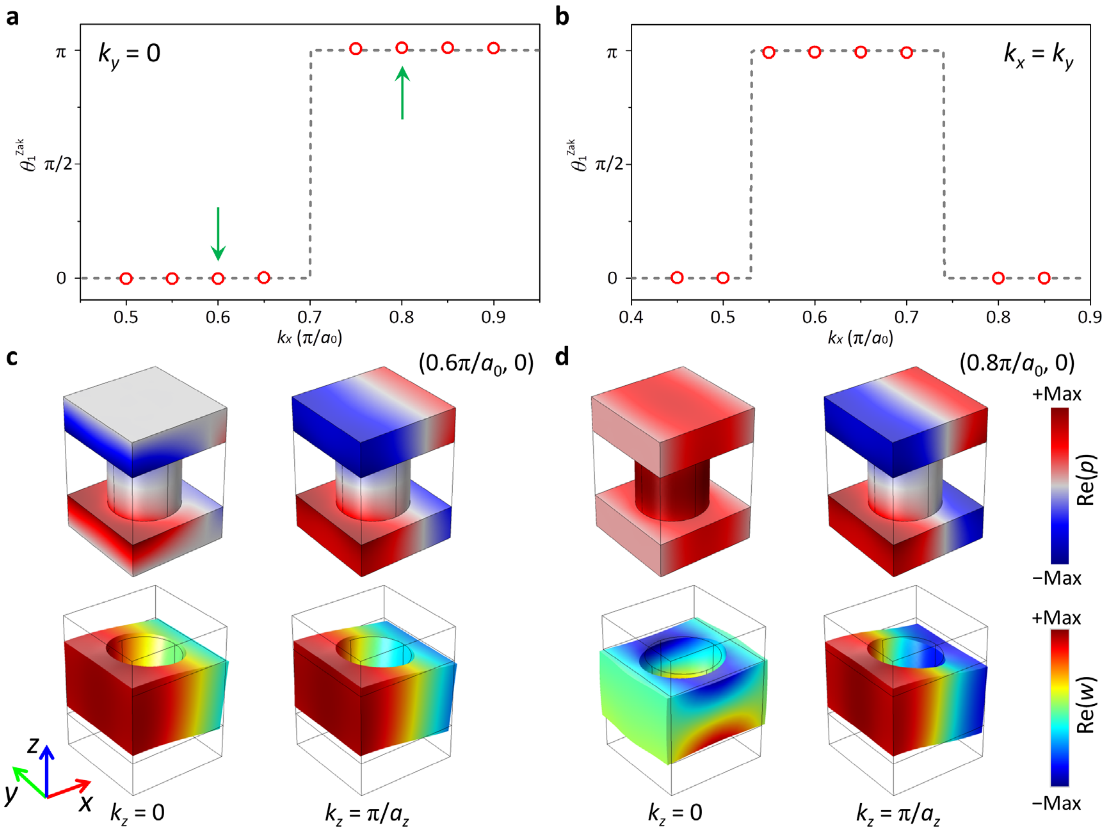

FIG. S10. Numerical calculation of Zak phases. (a),(b) Numerically calculated Zak phases along the line $k_y = 0$ (a) and $k_x = k_y$ (b) in the surface FBZ. The dots represent numerical values calculated from full-wave simulations, and dashed lines represent theoretical values obtained from the distribution of nodal rings. (c),(d) Field maps of the eigenmodes of the 1st band at $k_z = 0$ and $k_z = \pi/a_z$, respectively, corresponding to $\mathbf{k}_r = (0.6\pi/a_0, 0)$ (c) and $\mathbf{k}_r = (0.8\pi/a_0, 0)$, indicated by arrows in (a). The parities of the eigenmodes under mirror symmetry M_z confirm that the Zak phase is 0 at $\mathbf{k}_r = (0.6\pi/a_0, 0)$ and π at $\mathbf{k}_r = (0.8\pi/a_0, 0)$. The thin solid lines outline the profile of the unit cells without elastic displacements.

Supplementary Note 11

Three-dimensional band structure of DSSs

The three-dimensional band structure of the DSSs is retrieved and plotted in Fig. S11.

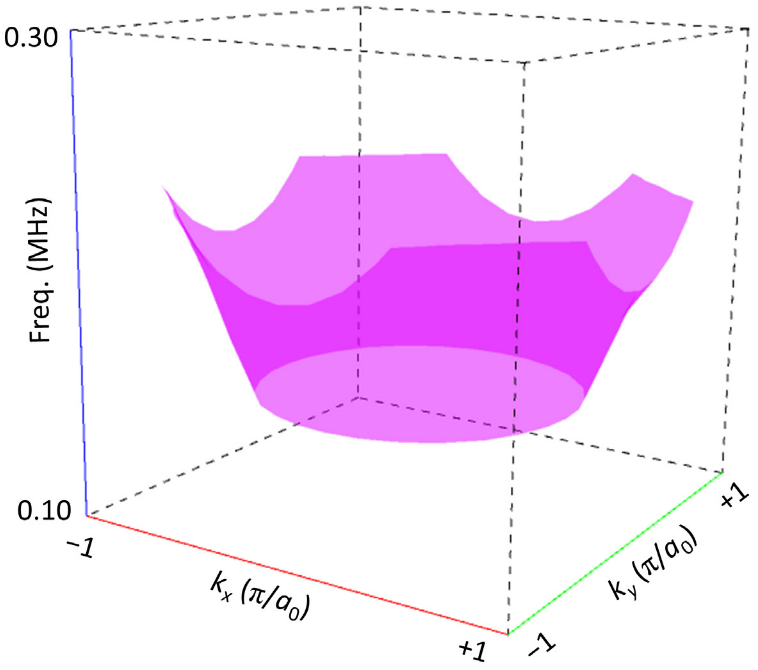

FIG. S11. Three-dimensional band structure of DSSs. The cyan surface represents the three-dimensional band structure of the DSSs for the supercell terminated by water.

Supplementary Note 12

Distribution of energy in supercell for DSSs

We plot the distribution of energy for the DSSs along inward direction of the supercell at $\mathbf{k}_r = (0.85\pi/a_0, 0)$ and $\mathbf{k}_r = (0.65\pi/a_0, 0.65\pi/a_0)$ in Fig. S12. The energies are calculated by integrals of energy densities in the water and aluminum plates, respectively, and the maximum is normalized to unity. It can be seen that the energies are both distributed in aluminum plates and water for the DSSs.

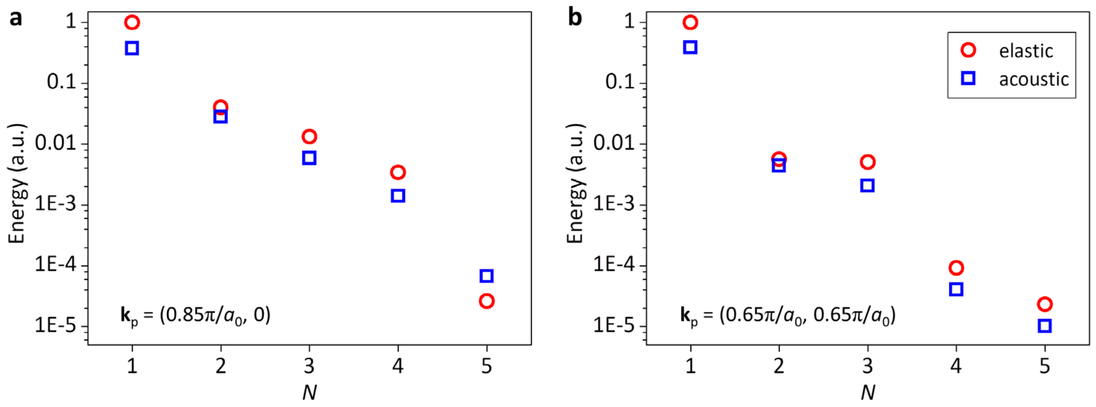

FIG. S12. Distribution of energy in supercell for DSSs. (a),(b) The red (blue) scatters represent the elastic (acoustic) energy in the aluminum (water) of the N-th unit cell counting from the surface (N = 1) when $\mathbf{k}_r$ = (0.85π/a_0, 0) (a) and $\mathbf{k}_r$ = (0.65π/a_0, 0.65π/a_0) (b). The maximum energy is normalized to unity.

Supplementary Note 13

Experimentally imaged field maps

The field maps at different frequencies that are experimentally imaged when we probe the surface states are plotted in Fig. S13.

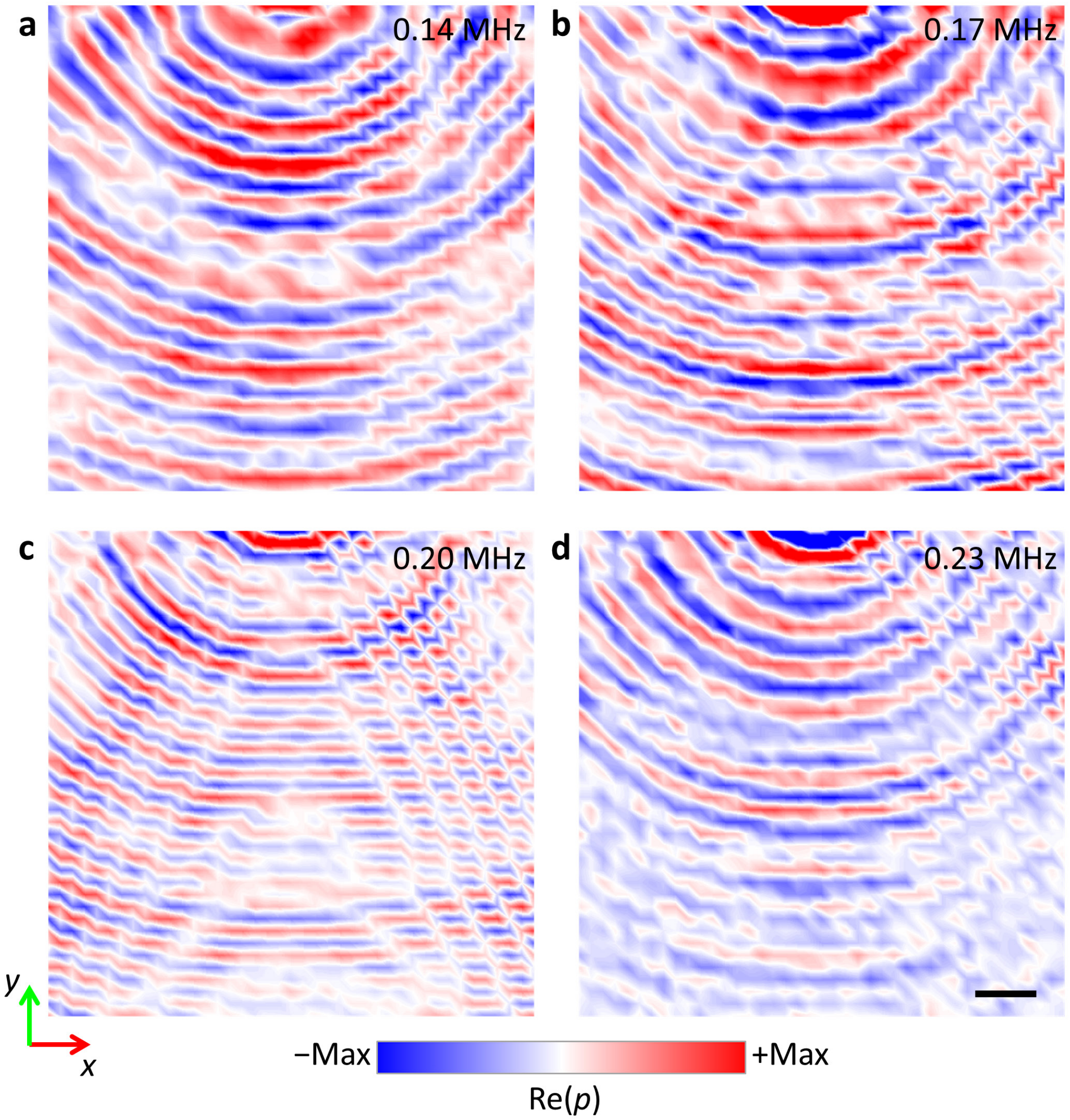

FIG. S13. Experimentally imaged field maps. (a)-(d) The experimentally imaged field maps when probing surface states, exciting at the frequency f = 0.14 MHz (a), 0.17 MHz (b), 0.20 MHz (b), and 0.23 MHz (d), respectively. The scale bar is 15 mm.

Supplementary Note 14

Type-II Weyl points from lowering symmetries

We lower the symmetry of the unit cell by adding two orthogonal through holes on the aluminum plates, as shown in Figs. S14(a) and Fig. S14(b). The geometric

parameters are t_m = 3.0 mm, t_w = 2.0 mm, d_0 = 1.6 mm, d_1 = 1.0 mm, and δ_z = 0.9 mm. The calculated band structure shown in Fig. S14(c) confirm that the red nodal ring is now broken, giving rise to two pairs of type-II Weyl points at the diagonals of k_z = 0 plane.

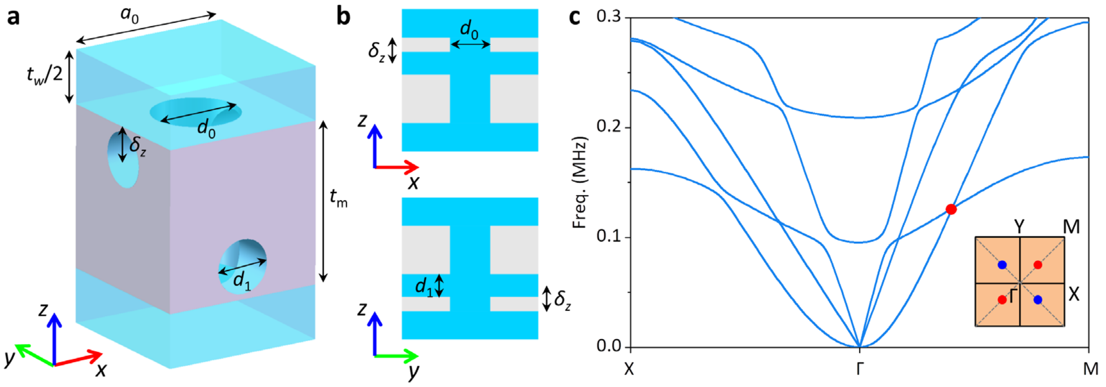

FIG. S14. Type-II Weyl points from lowering symmetries. (a) Schematic of the unit cell with lower symmetries. (b) Sectional schematics of the unit cell. (c) The calculated band structure on k_z = 0. Inset shows the distribution of type-II Weyl points of opposite charges on k_z = 0 plane.

Supplementary Note 15

Opposite parities of WG and FL modes

To demonstrate the opposite parities of the WG and FL modes with respect to the mirror symmetry M_z ($z \to -z$) when k_z = 0, we plot their field maps at two different random points on the k_z = 0 plane. As demonstrate in Fig. S15, one is $\mathbf{k} = (0.22\pi/a_0, 0.39\pi/a_0, 0)$ which is inside the red nodal ring, the other is $\mathbf{k} = (0.78\pi/a_0, -0.34\pi/a_0, 0)$ which is outside the red nodal ring. The modes of the phononic crystal are comprised of the scalar pressure field $p(x, y, z)$ in the fluid and the vectorial displacement field

u(x, y, z) in the solid. For the pressure field $p(x, y, z)$, we have

$$M_z p(x,y,z) = p(x,y,-z)\,, \tag{S36}$$

while for the displacement field **u**$(x, y, z) = (u(x, y, z), v(x, y, z), w(x, y, z))$, we have

$$\begin{aligned} M_z u(x,y,z) &= u(x,y,-z) \\ M_z v(x,y,z) &= v(x,y,-z) \\ M_z w(x,y,z) &= -w(x,y,-z) \end{aligned}\;. \tag{S37}$$

From the field maps demonstrated Fig. R7, we can see that, for the FL mode,

$$\begin{aligned} p(x,y,-z) &= -p(x,y,z) \\ u(x,y,z) &= -u(x,y,-z) \\ v(x,y,z) &= -v(x,y,-z) \\ w(x,y,z) &= w(x,y,-z) \end{aligned}\,, \tag{S38}$$

while for the WG mode

$$\begin{aligned} p(x,y,-z) &= p(x,y,z) \\ u(x,y,z) &= -u(x,y,-z) \\ v(x,y,z) &= -v(x,y,-z) \\ w(x,y,z) &= -w(x,y,-z) \end{aligned}\;. \tag{S39}$$

Hence for the FL mode, we have

$$\begin{aligned} M_z p(x,y,z) &= p(x,y,-z) = -p(x,y,z) \\ M_z u(x,y,z) &= u(x,y,-z) = -u(x,y,z) \\ M_z v(x,y,z) &= v(x,y,-z) = -v(x,y,z) \\ M_z w(x,y,z) &= -w(x,y,-z) = -w(x,y,z) \end{aligned} \tag{S40}$$

that is, the parity of the FL mode under M_z is −1. In contrast, for the WG mode, we have

$$\begin{aligned} M_z p(x,y,z) &= p(x,y,-z) = p(x,y,z) \\ M_z u(x,y,z) &= u(x,y,-z) = u(x,y,z) \\ M_z v(x,y,z) &= v(x,y,-z) = v(x,y,z) \\ M_z w(x,y,z) &= -w(x,y,-z) = w(x,y,z) \end{aligned} \tag{S41}$$

that is, its parity under M_z is +1. Therefore, the FL mode and WG mode correspond to the two different eigenmodes of the mirror symmetry $M_z(z \to -z)$, which prevents

their coupling when $k_z = 0$.

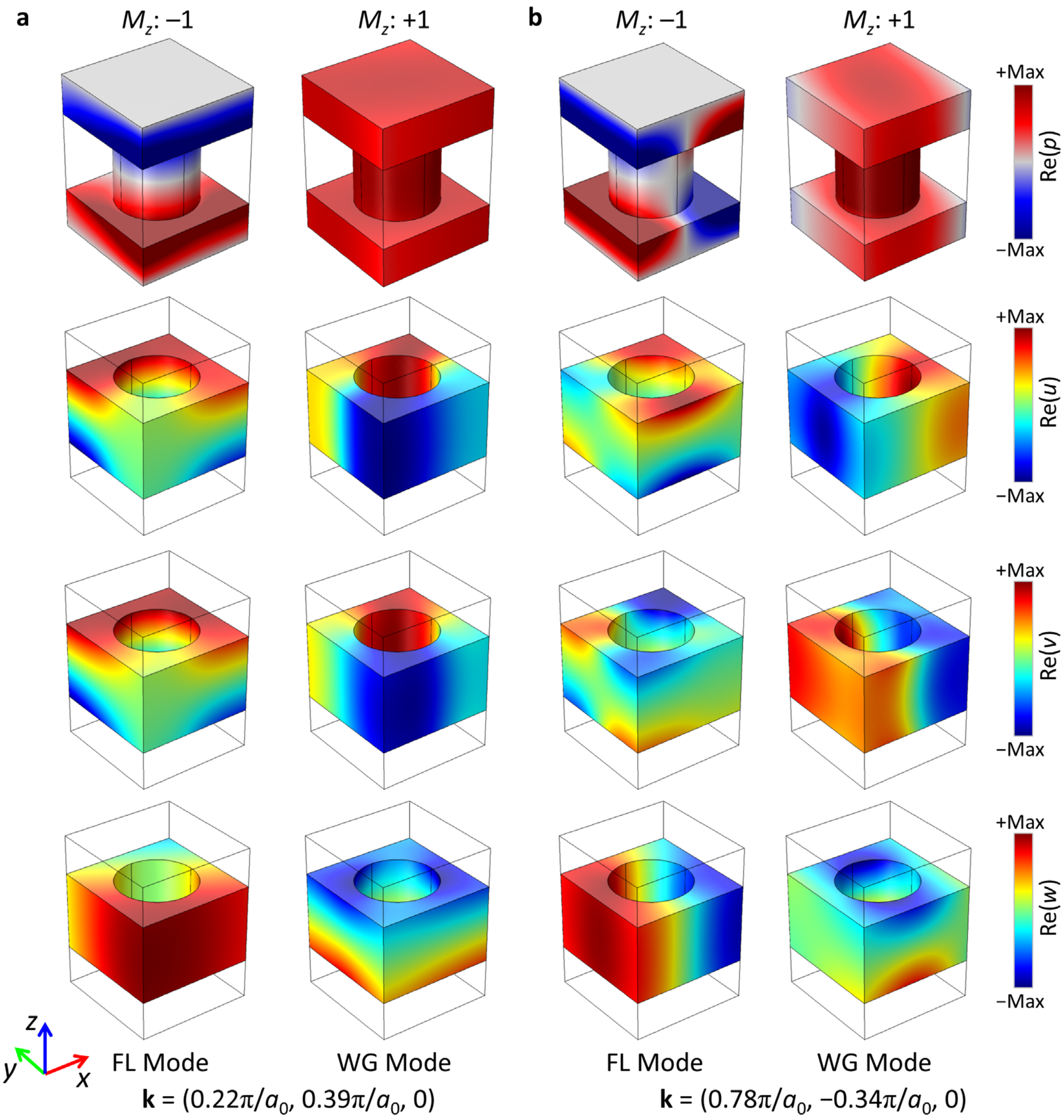

FIG. S15. Parities of mirror symmetry for FL and WG modes. (a),(b) Field maps of the FL and WG modes when **k** = (0.22π/a_0, 0.39π/a_0, 0) which is inside the red nodal ring (a) and when **k** = (0.78π/a_0, −0.34π/a_0, 0) which is outside the red nodal ring (b). Both the fields of acoustic pressure (Re(p)) and elastic displacement (Re(u), Re(v), Re(w)) are shown. The parity with respect to the mirror symmetry $M_z(z \to -z)$ is −1 for the FL mode and +1 for the WG mode.

Supplementary Note 16

Fluid-solid interaction and topological phase

We consider the case of stacked metallic plates with blind holes immersed in water. We start by modeling the aluminum plates with pressure acoustics only, the same way as water. The fluid-solid interaction won't come into play, and we simply employ the density ρ = 2700 kg/m^3 and sound of speed c = 6300 m/s for aluminum. The calculated band structure is shown in Fig. S16(a), and it can be seen that only one mode emerges from Γ point, and there is no nodal ring for the first band and no sign of topological acoustic effects.

In comparison, we include the fluid-solid interaction properly in our work. The calculated band structure in our way, as demonstrated in Fig. S16(b), shows that the red nodal ring is formed between the 1st and 2nd bands due to the FL mode and the WG mode. The FL mode can only exist if we consider the shear sound in the metallic plates, ignored in these mentioned works. Furthermore, we consider a supercell of the phononic crystal terminated by the *x*-*y* surface to further illuminate the importance of the fluid-solid interaction for the topological effects in our work. When we do not include the fluid-solid interaction and model both the aluminum and water with pressure acoustics, from the calculated projected band structure demonstrated in Fig. S16(c), we can see that there is indeed no topological effect. On the contrary, after we include the fluid-solid interaction, the strongly tilted drumhead surface state (DSS) emerges in the partial bandgap as demonstrated in Fig. S16(d).

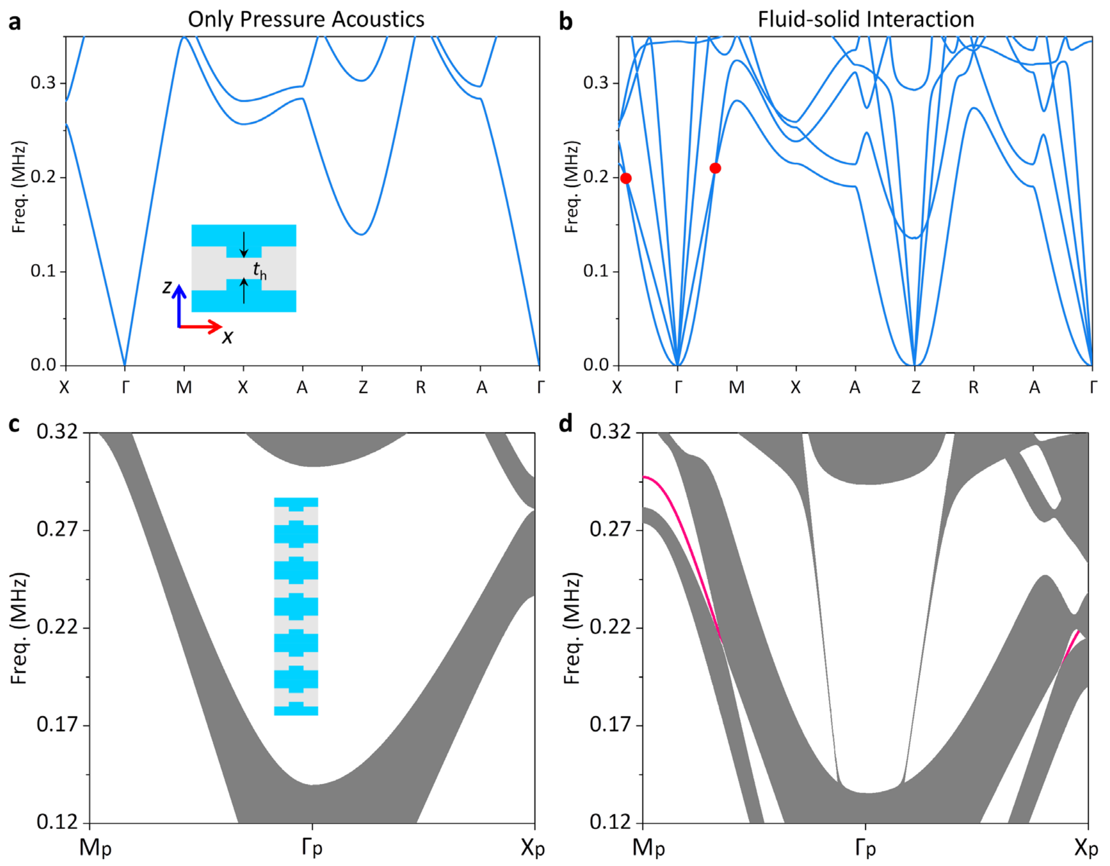

FIG. S16. Band structures versus fluid-solid interaction. (a) Calculated Band structure of the phononic crystal without fluid-solid interaction. The phononic crystal is comprised of stacked aluminum plates with blind holes immersed in water (Fig. 2(d) in the main text). The inset shows the schematic of the unit cell. Specifically, the separation between holes on the two sides of the plates t_h = 1.0 mm. (b) Calculated band structure of the same phononic crystal with fluid-solid interaction. The red nodal ring is formed between the waveguide (WG) mode and the flexural Lamb (FL) mode. (c) Calculated projected band structure of the supercell terminated by *x*-*y* surface without fluid-solid interaction, corresponding to (a). The inset shows the schematic of the supercell. (d) Calculated projected band structure of the supercell terminated by *x*-*y* surface with fluid-solid interaction, corresponding to (b). Gray shaded regions denote the projected bulk bands. Solid lines represent the strongly

tilted drumhead surface state.

Supplementary Note 17

Origin of blue and green nodal rings

The perforated holes are critical for the emergence of other nodal rings except the red one. To confirm this point, we have calculated the bands of the phononic crystal with the metallic plate assumed to be acoustically rigid. The calculated band structure is shown in Fig. S17(a), and it can be seen that only the blue nodal ring on $k_z = \pi/a_z$ exists, as demonstrated in Fig. S17(b). On the other hand, the red nodal ring arising from the fluid-solid interaction is missing because the plates are now acoustically rigid and cannot support flexural Lamb modes. The results suggest that the blue nodal ring on $k_z = \pi/a_z$ is due to the degeneracy between the waveguide (WG) mode and the acoustic resonance mode of the through holes. Both modes still exist when the plates are acoustically rigid. To demonstrate this point, we plot the field maps of the two modes around the blue nodal ring at $\mathbf{k} = (0.83\pi/a_0, 0.75\pi/a_0, \pi/a_z)$. As shown in Fig. S17(c), the two modes indeed have opposite parities with respect to the mirror symmetry M_z, as expected. On the other hand, if the holes are blind holes on the rigid plates, for example, with a separation $t_\mathrm{h} = 1.0$ mm between holes on two sides of the plates, we can see that the blue nodal rings will disappear, as shown in Fig. S17(d). This fact is because if the holes are blind holes, the frequency of their first-order acoustic resonance will be significantly increased because the effective length of the holes are greatly reduced.

In other words, we can effectively tune the blue nodal rings by tuning the acoustic resonance mode, through changing the thickness of the plates t_m. For example, we consider the case that the holes are through holes, and the thickness of rigid plates is t_m = 1.0 mm, while other geometric parameters remain unchanged. The calculated band structure is shown in Fig. S17(e). The blue nodal rings expand and reconnect after touching each other, now centered around R point in the reciprocal space, as demonstrated in Fig. S17(f). The green nodal rings now also appear on $k_x = \pi/a_0$ and $k_y = \pi/a_0$ planes, connected with the blue nodal rings. In fact, the existence of green nodal rings, when the blue nodal rings are centered around R point, is guaranteed by the mirror symmetries with respect to the $k_x = \pi/a_0$ and $k_y = \pi/a_0$ planes in the reciprocal space. The mirror symmetries lead to the opposite orientations of the blue nodal rings on opposite sides of the $k_x = \pi/a_0$ and $k_y = \pi/a_0$ planes [5].

In summary, the through holes introduce an acoustic resonance mode, which has opposite parities with the WG mode when $k_z = \pi/a_z$, and they can form nodal rings on the high-symmetry planes in the reciprocal space. In contrast, if the holes are blind holes, because the resonance modes of the holes are significantly shifted towards higher frequencies, there will be no nodal rings when $k_z = \pi/a_z$.

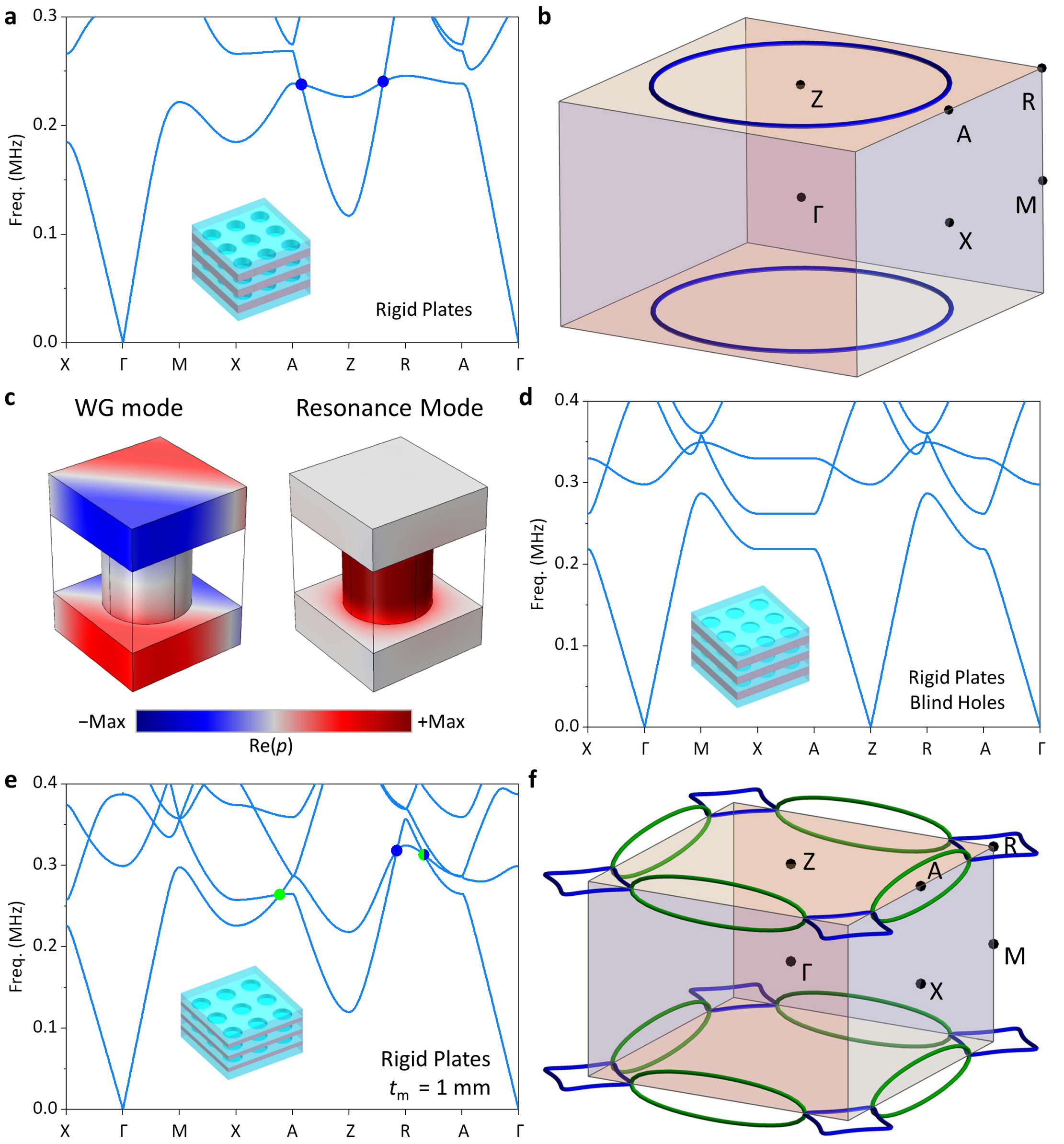

FIG. S17. Band structures when the plates are rigid. (a) Calculated band structure of the unit cell when its solid domain is acoustically rigid, while geometric parameters are all the same. The inset shows the schematic of the unit cell. (b) Distribution of the nodal ring. (c) The field maps of the first two bands around the blue nodal ring, specifically, $\mathbf{k}$ = ($0.83\pi/a_0$, $0.75\pi/a_0$, π/a_z). The two modes are a waveguide (WG) mode and an acoustic resonance mode. (d) Calculated band structure of the unit cell when the holes are blind holes. Specifically, the separation between the holes on the two sides of the rigid plates is t_h = 1.0 mm (the same as in Fig. S5(a) in

Supplementary Information). The inset shows the schematic of the unit cell. There are no nodal rings now. (e) Calculated band structure when the thickness of rigid plates t_m = 1.0 mm. The inset shows schematic of the unit cell. (f) Distribution of the nodal rings. The green nodal rings emerge and connect with the blue nodal rings.

Supplementary Note 18

Interface state by breaking mirror symmetry

Here, we show how to generate interface states in phononics by breaking the type-II nodal ring, which can be considered as a 3D extension of the valley-Hall kink states. As demonstrated in Fig. S18(a), now we employ stacked metallic plates with blind holes, and the blind holes are only on one side of the plates which breaks the mirror symmetry M_z. In this structure, the red nodal ring protected by M_z is then gapped as indicated by the red arrow. By combining the plates with blind holes on opposite sides as displayed in Fig. S18(b), the calculated projected band structure of the supercell reveals that a state emerges in the partial bandgap between the 1st and 2nd bulk bands, as shown in Fig. S18(c). The field maps in Fig. S18(d) confirm that the state in the partial bandgap is indeed localized at the interface between the two different domains. The interface state is in fact due to the distinct topological properties of the two domains characterized by opposite distributions of the Berry curvature around the gapped nodal ring [6], originating from their different ways to break the mirror symmetry M_z.

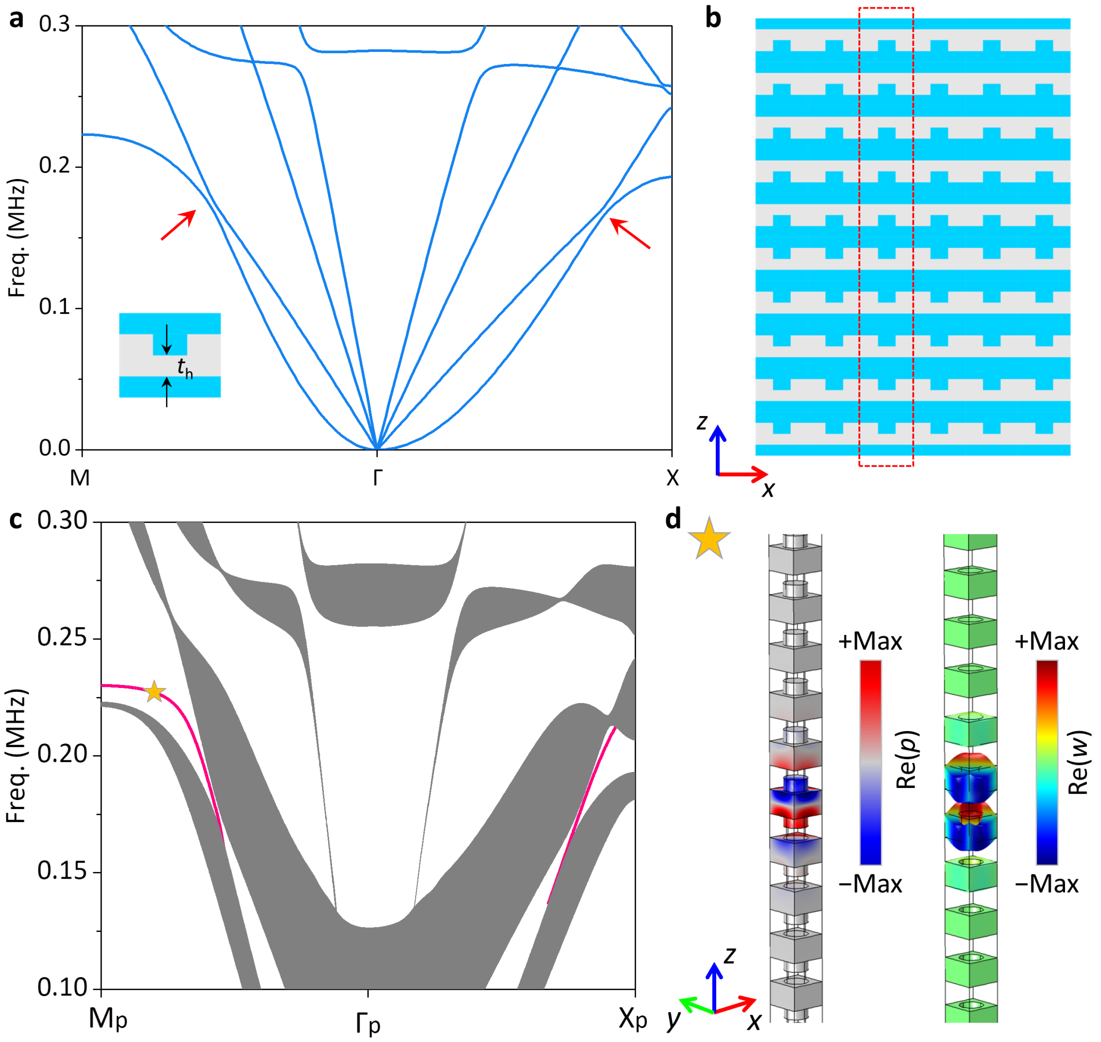

FIG. S18. Interface state from symmetry breaking. (a) Calculated band structure along high-symmetry directions for the unit cell with blind holes on one side of the plates. Inset: schematic of the unit cell, specifically, we choose t_h = 1.0 mm. The red nodal ring is gapped as indicated by the red arrows. (b) Schematic of the supercell, which is comprised of the unit cells with blind holes on opposite sides. (c) Projected band structure of the supercell. Solid lines represent the interface state. Gray shaded regions denote the projected bulk bands. (d) Calculated field maps of acoustic pressure (Re(p)) and elastic displacements (Re(u), Re(v), Re(w)) for the marked point in (c).

Supplementary Note 19

Bulk state at low frequency range when probing surface states

The states that emerge at low frequency range in the Fourier spectra when probing surface states are bulk states rather than surface states. It is because in experiments, we cannot prevent the excitation of bulk states. To confirm this point, we have performed simulations with a source to excite the phononic crystal structure, and the retrieved Fourier spectra are shown in Fig. S19(a). It can be seen that the bright stripes also emerge at low frequencies, just as they do in the experimental results (Fig. 4(d) in the main text), which is also shown here as Fig. S19(b). We would like to mention that, because of the limited RAM of our workstation (512 GB), we can only model 10 layers of the metallic plates with an area of 20×20 periods in the simulations, instead of the real experiment setup (10 layers, 100×100 periods). As a result, the simulations do not correspond exactly to the experiments. Nevertheless, it indicates that the "extra surface states" emerging at low frequency range are excited bulk states rather than surface states. The acoustic field maps when the phononic crystals are excited at 0.12 MHz and 0.16 MHz are demonstrated in Figs. S19(c) and S19(d), respectively. It is clear that whether or not there are drumhead surface states, modes are excited in the structure, which are represented by bright stripes in the retrieved Fourier spectra.

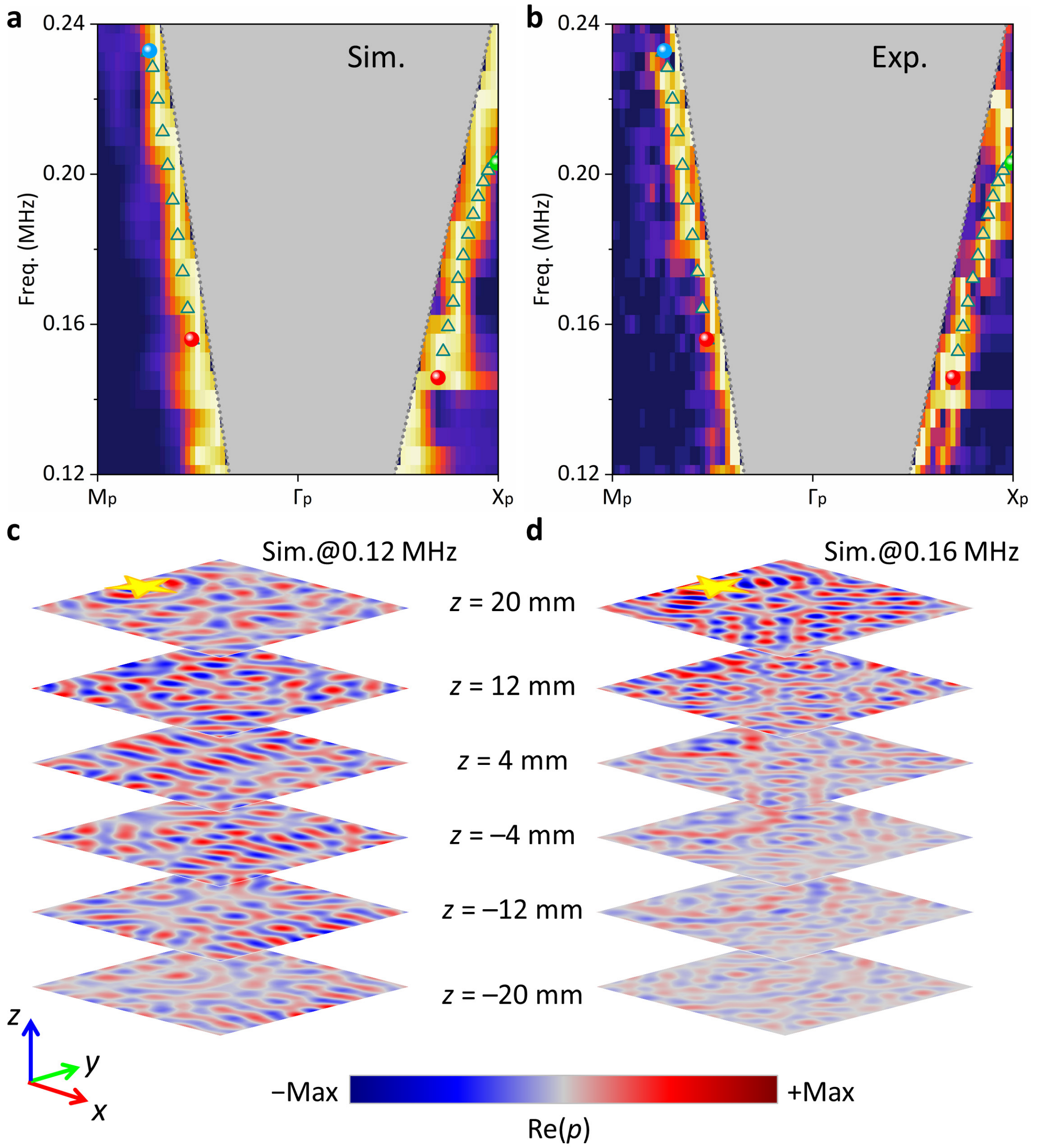

FIG. S19. Emergence of states at low frequency range. (a) Retrieved Fourier spectra from simulations. (b) Retrieved Fourier spectra from experiments (Fig. 4(d) in the main text) for comparison. (c),(d) Simulated field maps at 0.12 MHz (c) and 0.16 MHz (d). The star represents where we place the source to excite the structure.

Supplementary Note 20

Control experiment with lattice constant 4 mm

We change the in-plane lattice constant of the perforated holes to a_0 = 4 mm and carried out additional experiments on the new control sample. Other geometric parameters are kept unchanged. The calculated band structure along high-symmetry directions is shown in Fig. S20(a). The photographs of the new control sample is shown in Fig. S20(b). The new Fourier spectra experimentally retrieved along high-symmetry directions for bulk bands is shown in Fig. S20(c). For the new sample, good agreement is observed between the bright stripes in the experimental Fourier spectra and the calculated bulk bands projected along k_z direction. For comparison, the retrieved Fourier spectra when a_0 = 3 mm in the main text is also demonstrated here as Fig. S20(d). From the shift of the bright stripes which generally overlap with the projected bulk modes calculated from full-wave simulations, we can conclude that the observed signals are indeed owing to the bulk bands of the phononic crystals.

We can consider about the DSSs. The projected band structure and the retrieved Fourier spectra for detection of surface states when a_0 = 4 mm are shown in Figs. S21(a) and Fig. S21(b), respectively. For comparison, the results for a_0 = 3 mm in the main text are also demonstrated here as Figs. S21(c) and S21(d), respectively. In both cases, good agreement is observed between the bright stripes in the experimentally retrieved Fourier spectra and the numerically calculated dispersion of the drumhead surface states in both cases. The comparison between the experimentally retrieved Fourier spectra for different lattice constants also confirms that the detected signal includes contribution from the excited drumhead surface state. The imaged field maps at selected frequencies for probing the surface states when a_0 = 4 mm are shown as

Fig. S22.

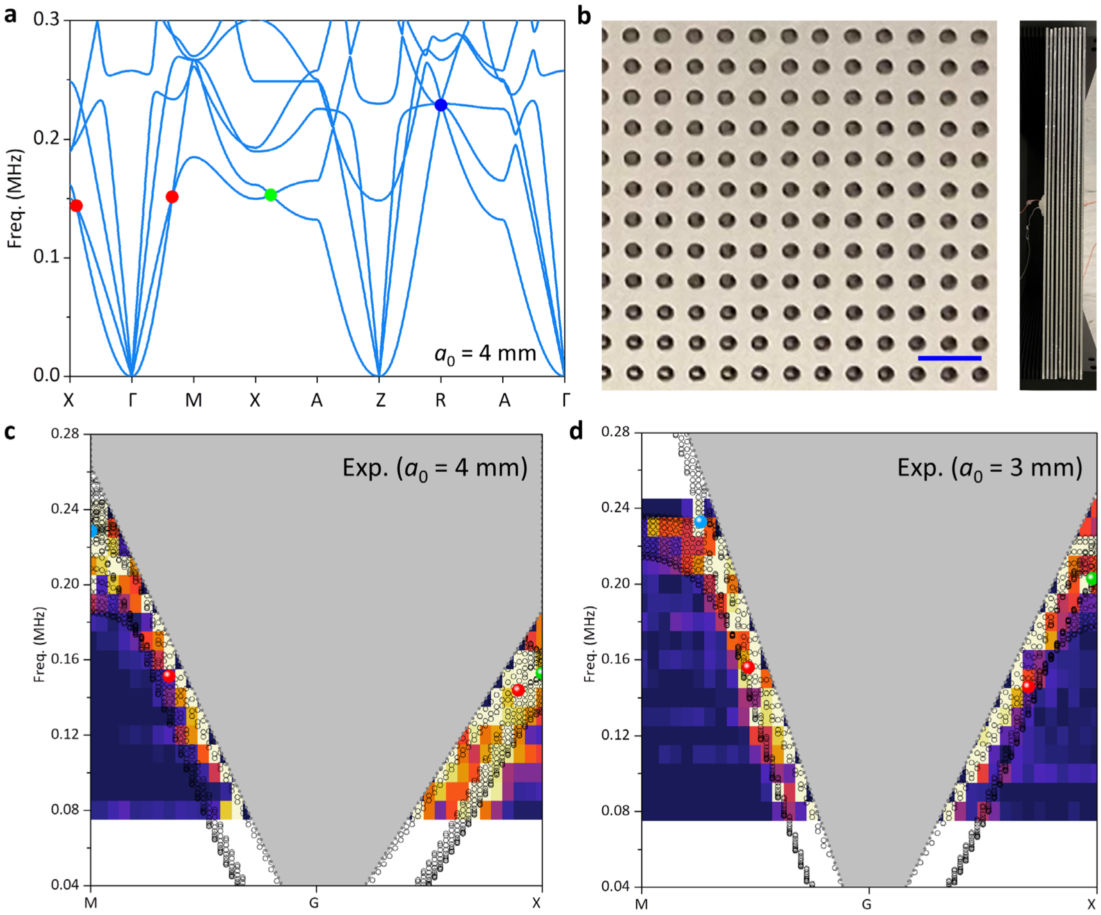

FIG. S20. Experimentally retrieved Fourier spectra for bulk bands. (a) Calculated band structure when the in-plane lattice constant a_0 = 4 mm. (b) Photographs of the control sample with a_0 = 4 mm. Left panel: close view of the perforated holes. Blue scale bar: 8 mm. Right panel: top view. (c) Experimentally retrieved Fourier spectra along high-symmetry directions when a_0 = 4 mm. Black circles: calculated band structure projected along k_z. The colored dots represent the nodal rings of the same color denoted in (a). (d) Experimentally retrieved Fourier spectra along high-symmetry directions when a_0 = 3 mm for comparison, essentially the same as Fig. 3(d) in the main text.

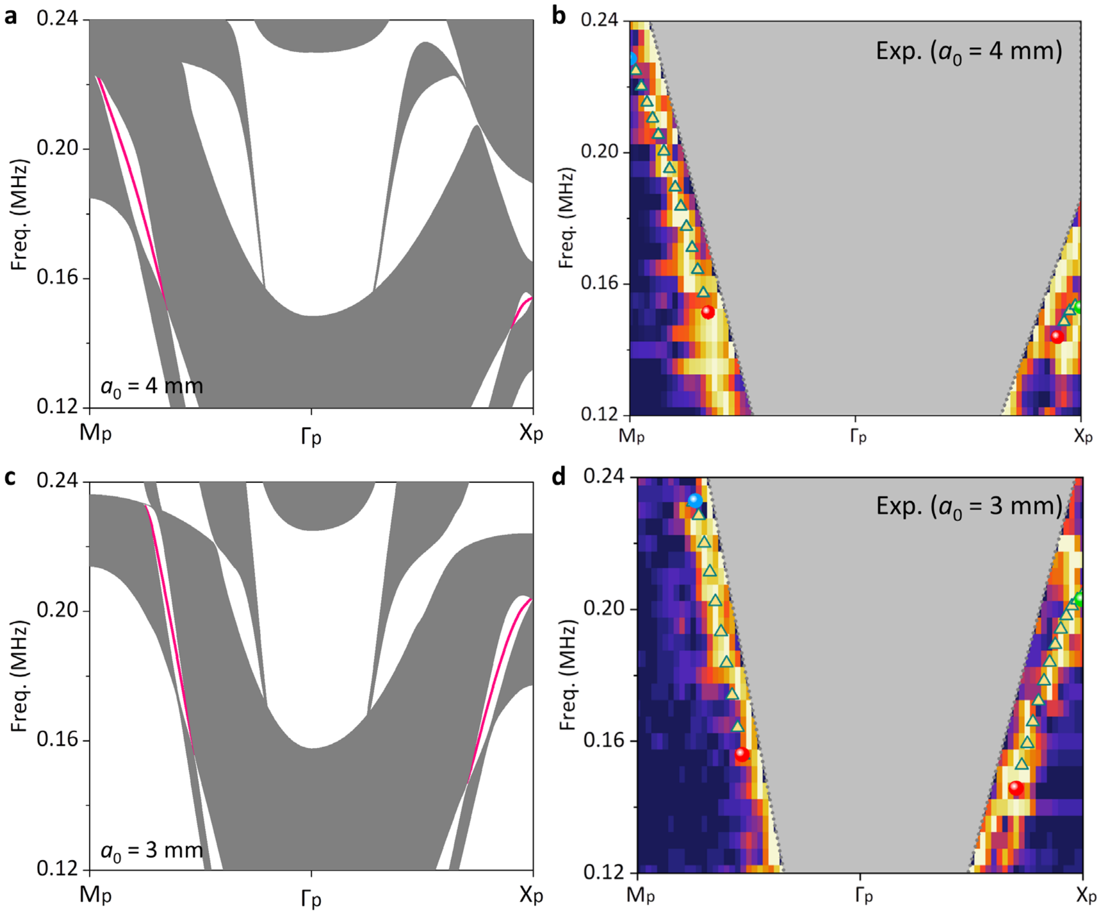

FIG. S21. Experimentally retrieved Fourier spectra for surface states. (a) Projected band structure for the supercell with a_0 = 4 mm. (b) Experimentally retrieved Fourier spectra with a_0 = 4 mm when probing surface states. (c) Projected band structure for the supercell with a_0 = 3 mm for comparison. (d) Experimentally retrieved Fourier spectra with a_0 = 3 mm for comparison when probing surface states, essentially the same as Fig. 4(d) in the main text.

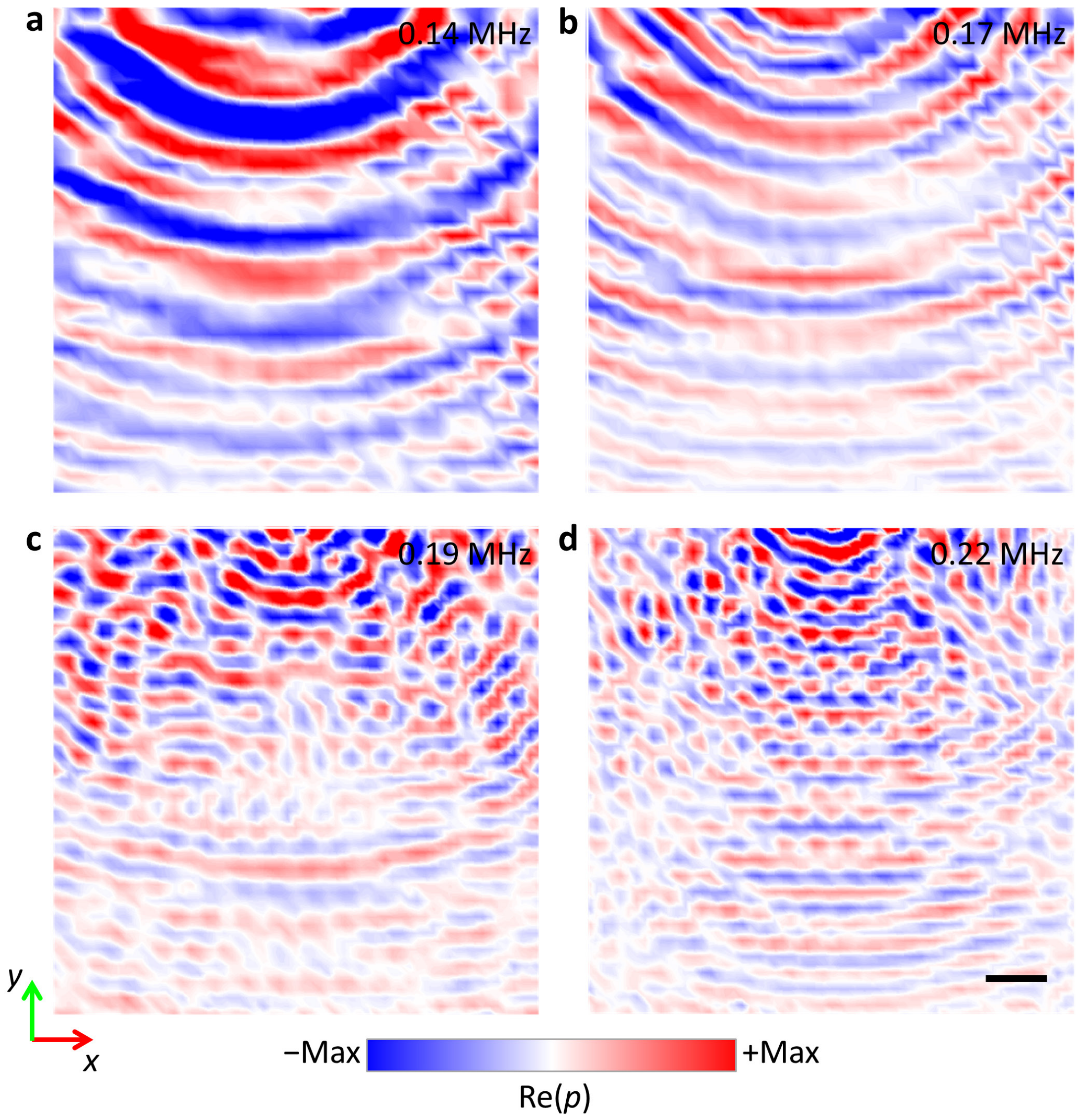

FIG. S22. Experimentally imaged field maps. (a)-(d) The experimentally imaged field maps when probing surface states with lattice constant a_0 = 4 mm, exciting at the frequency f = 0.14 MHz (a), 0.17 MHz (b), 0.19 MHz (b), and 0.22 MHz (d), respectively. The scale bar is 15 mm.

References

[1] I. V. Shadrivov, A. A. Sukhorukov, and Y. S. Kivshar, Complete band gaps in one-dimensional left-handed periodic structures, Physical Review Letters **95**, 193903 (2005).

[2] D. A. Kiefer, M. Ponschab, S. J. Rupitsch, and M. Mayle, Calculating the full

leaky Lamb wave spectrum with exact fluid interaction, The Journal of the Acoustical Society of America **145**, 3341 (2019).

[3] Z. Xiong, H.-X. Wang, H. Ge, J. Shi, J. Luo, Y. Lai, M.-H. Lu, and J.-H. Jiang, Topological node lines in mechanical metacrystals, Physical Review B **97**, 180101 (2018).

[4] H.-X. Wang, G.-Y. Guo, and J.-H. Jiang, Band topology in classical waves: Wilson-loop approach to topological numbers and fragile topology, New Journal of Physics **21**, 093029 (2019).

[5] E. Yang, B. Yang, O. You, H.-C. Chan, P. Mao, Q. Guo, S. Ma, L. Xia, D. Fan, and Y. Xiang, Observation of non-Abelian nodal links in photonics, Physical Review Letters **125**, 033901 (2020).

[6] B. Yang, Y. Bi, R.-X. Zhang, R.-Y. Zhang, O. You, Z. Zhu, J. Feng, H. Sun, C. T. Chan, and C.-X. Liu, Momentum space toroidal moment in a photonic metamaterial, Nature Communications **12**, 1784 (2021).
